# Food Polyphenols Fail to Cause a Biologically Relevant Reduction of COX-2 Activity

**DOI:** 10.1371/journal.pone.0139147

**Published:** 2015-10-06

**Authors:** Ina Willenberg, Anna K. Meschede, Faikah Gueler, Mi-Sun Jang, Nelli Shushakova, Nils Helge Schebb

**Affiliations:** 1 University of Veterinary Medicine Hannover, Institute for Food Toxicology and Analytical Chemistry, Bischofsholer Damm 15, 30173 Hannover, Germany; 2 Department of Nephrology, Hannover Medical School, Carl-Neuberg-Str. 1, 30625 Hannover, Germany; 3 Phenos GmbH, Feodor-Lynen-Straße 5, 30625 Hannover, Germany; 4 University of Wuppertal, Institute of Food Chemistry, Gaußstraße 20, 42119 Wuppertal, Germany; Universidade de São Paulo, BRAZIL

## Abstract

Epidemiologic studies show a correlation between the dietary intake of food polyphenols and beneficial health effects. Several *in vitro* studies indicate that the anti-inflammatory potential of polyphenols is, at least in part, mediated by a modulation of the enzymes of the arachidonic acid cascade, such as the prostaglandin forming cyclooxygenases (COXs). Evidence that this mode of action can be transferred to the situation *in vivo* is scarce. This study characterized effects of a subset of polyphenols on COX–2 expression and activity *in vitro* and compared the potency with known drugs. Next, the *in vivo* relevance of the observed *in vitro* effects was tested. Enzyme assays and incubations of polyphenols with the cancer cell line HCA–7 and lipopolysaccharide (LPS) stimulated primary monocytes support the hypothesis that polyphenols can effect COX–2 expression and activity *in vitro*. The effects were most pronounced in the monocyte assay for wogonin, apigenin, resveratrol and genistein with IC_50_ values of 1.5 μM, 2.6 μM, 2.8 μM and 7.4 μM. However, these values are 100- to 1000-fold higher in comparison to those of the known pharmaceuticals celecoxib, indomethacin and dexamethasone. In an animal model of LPS induced sepsis, pretreatment with polyphenols (i. p. 100 mg/kg bw) did not result in decreased plasma or tissue prostaglandin levels, whereas the positive control celecoxib effectively attenuated LPS induced prostaglandin formation. These data suggest that despite the moderate potency *in vitro*, an effect of polyphenols on COX–2 during acute inflammation is unlikely, even if a high dose of polyphenols is ingested.

## Introduction

The dietary intake of fruits and vegetables is correlated with a longer healthier life. Health promoting effects are discussed for secondary plant metabolites in particular for polyphenols: Epidemiological studies suggest beneficial effects of polyphenols on cardiovascular diseases and the risk for the development of cancer [1, 2]. Chronic inflammation plays an important role in the development of these diseases and a large number of studies report anti-inflammatory effects for polyphenols [3, 4]. Aside from their antioxidative and radical scavenging properties, a modulation of pro-inflammatory mediators formed in the arachidonic acid (AA) cascade, such as cyclooxygenase–2 (COX–2) products, are suggested as modes of action underlying the anti-inflammatory effects [3]. COX–2 is expressed during inflammatory processes, giving rise to a large number of biological active prostaglandins (PGs), for example the pain, fever and inflammation mediating PGE_2_ [5]. Based on this central role COX–2 is a major target of selective COX–2 inhibitors (COX-2i) and non-steroidal anti-inflammatory drugs (NSAIDs), which also inhibit the constitutively expressed COX–1. Effects of polyphenols on COX–2 activity, have been demonstrated in a vast number of *in vitro* studies [6–11]. However, different test systems were used, and the lack of a correlation with the efficacy of known COX-2i or NSAIDs makes it impossible to compare and evaluate the potency of polyphenols. Moreover, a highly potent inhibition of COX–2 *in vitro* does not directly translate into an anti-inflammatory potential *in vivo*. The aim of the present study was to evaluate if food polyphenols could elicit a pharmacological relevant inhibition of COX–2. Therefore polyphenols, which are known to inhibit COX–2 *in vitro*, i.e. nobiletin [9, 12], naringenin [7, 13], apigenin [6, 10, 13], wogonin [8, 14], genistein [10], epigallocatechingallat (EGCG) [10, 15] and resveratrol [11, 16, 17], as well as the resveratrol oligomers ε-viniferin and hopeaphenol, were comprehensively analyzed regarding their effects on COX–2. In a tiered approach the effect on COX–2 was tested in a cell-free enzyme assay, in a cancer cell line and in lipopolysaccharide (LPS) stimulated primary human monocytes. Finally, the most potent COX–2 inhibitors were tested for their ability to modulate COX–2 activity during acute inflammation in the LPS induced sepsis model with multi organ failure. In this model a modulation of all branches of the AA cascade by polyphenols was analyzed by means of targeted metabolomics in plasma and different organs.

## Materials and Methods

### Chemicals

Resveratrol (≥ 99%), apigenin (≥ 97%), genistein (≥ 98%), naringenin (≥ 95%), EGCG (≥ 95%) and wogonin (≥ 98%) were purchased from Sigma (Schnellendorf, Germany). Hopeaphenol (≥ 90%) and ε-viniferin (≥ 90%) were obtained from Actichem (Montauban, France). Celecoxib was purchased from Santa Cruz Biotechnology (Dallas, USA).

### *In vitro* assays

The *in vitro* assays were performed as described [6, 16, 18] and COX metabolites were quantified by LC-MS [18].

In the cell-free assay polyphenols were incubated with ovine recombinant COX–1 (70 ng/mL, Cayman Chemicals/ Biomol, Hamburg, Germany) or human recombinant COX–2 (50 ng/mL, Cayman Chemicals).

HCA–7 cells were incubated with sub-cytotoxic concentrations of the polyphenols dissolved in Tris buffered DMEM medium. Cytotoxicity of the polyphenols was evaluated by the lactate dehydrogenase leakage test (Cyto-Tox-ONE, Promega Mannheim, Germany). After 24 h the supernatant was collected for LC-MS analysis and the cells were harvested to analyze the COX–2 expression by Western Blotting.

Primary human monocytes were freshly isolated from human blood. Blood was collected from healthy human subjects after written informed consent was obtained. The study was approved by the ethics committee of the Medical University Hannover (Nr. 6379, 11/2013). Monocytes were separated by gradient density centrifugation [18] and incubated with media containing 10 μg/mL LPS from *Escherichia coli* 0111:B4 (Sigma, Schnellendorf, Germany, L2630) and sub-cytotoxic concentrations of the polyphenols. After 24 h, the supernatant was sampled for LC-MS and cells were collected for the COX–2 expression analysis by Western Blotting. In all assays, the concentrations of the polyphenols were stable (recovery > 80%) for up to 24 h, as determined by LC. Inhibitory effects were calculated based on the PGE_2_ formation determined by LC-MS.

### Animals

Twelve week old male C57BL/6N mice (Charles River, Sulzfeld, Germany) were used for all experiments. C57BL/6 male mice (H2^b^, 11–13 weeks of age) were obtained from Charles River (Sulzfeld, Germany). Animals were cared for in accordance with the institution’s guidelines for experimental animals and with the guidelines of the American Physiological Society. The animal protection committee of the local authorities (Lower Saxony state department for food safety and animal welfare LAVES) approved all experiments (approval: 33.9-42502-04-12/0846). Mice were housed under conventional conditions in individually ventilated cages produced by Techniplast Inc. (Italy) with a 12 h light/dark cycle and had free access to food (Altromin 1324 standard mouse diet) and domestic quality drinking water ad libitum. Mice were monitored closely and if they appeared compromised (i.e. inactivity, no intake of food or water) after compound or LPS injection the experiment was terminated.

### *In vivo* model

C57BL/6N mice were pretreated with the polyphenols (i. p., 100 mg/kg bw) or vehicle (80/20 (*v/v*) PEG 400/DMSO, 5μL/g bw). After 2 h mice were i. p. challenged with 10 mg/kg bw LPS (from *E*.*coli* 0111:B4, Sigma, L2630) or vehicle (10 μL/g bw). The control group, received COXi vehicle and LPS vehicle (n = 8), the LPS control group received the COXi vehicle and LPS (n = 7). Twenty-four hours after LPS treatment animals were sacrificed in general isofluran anesthesia by whole body perfusion with ice cold PBS. Thereafter, organ retrieval of liver and kidney samples was done and tissues were shock frozen and fixed in RNA later. Samples were stored at -80°C till further analysis. Blood drawing from the retro orbital venus plexus with an EDTA coated capillary was done at baseline (i.e. 4 days prior to study start) and 24 h thereafter. Plasma was generated by centrifugation at 4000 g at 4°C and stored at -80°C till further analysis.

### Oxylipin analysis

Oxylipin levels were analyzed in plasma and tissues by LC-MS following SPE extraction of 200 μl plasma or tissue homogenate (50 mg) on Chromabond C_18_ ec cartridges (Machery-Nagel, Düren, Germany) [19]. A list of the covered analytes can be found in the SI (S1 Table).

### Clinical chemistry

Clinical chemistry parameters (urea, creatinine, aspartate transaminase (AST), alanine transaminase (ALT), lactate dehydrogenase (LDH)) in plasma were analyzed at baseline (4 days prior the experiment) and 24 h after LPS injection. Parameters were determined by using the fully automated Olympus AU 400 analyzer (Beckman Coulter Inc.). Only healthy mice (AST below 80 U/ml and a LDH below 1000 U/ml) were included in the experiment.

### RNA extraction and real time quantitative PCR

Tissue sections were stored in RNA-later immediately after organ retrieval. Total RNA was extracted using the RNeasy mini kit system (Qiagen, Hilden, Germany) and transcribed using Superscript II Reverse transcriptase (Invitrogen). Quantitative (q) PCR was performed on Lightcycler 420 II (Roche Diagnostics, Penzberg, Germany) using FastStart Sybr-Green chemistry. Gene-specific primers for IL–6 (Quantitec QT00098875, Qiagen) and MCP–1 (Quantitec QT00167832, Qiagen) were used for the gene of interest and HPRT served as house keeping gene for normalization (Quantitec QT00166768, Qigaen). Quantification was carried out using qgene software.

### Data analysis

GraphPad Prism 5.0 (GraphPad Software, San Diego, USA) was used for data analysis, the fitting of dose response curves and calculation of the IC_50_ values. Statistical differences were determined by Dunnetts test (LPS vs. other groups).

## Results

### Enzyme assay

In the cell-free enzyme assay nobiletin, naringenin, wogonin and genistein showed no effect on COX–1 or COX–2 dependent PGE_2_ formation (Table 1). Incubation of COX–1 with 100 μM apigenin resulted in a 31% decreased PGE_2_ formation in comparison to the control, while the COX–2 activity remained unaffected. EGCG, resveratrol, ε-viniferin and hopeaphenol dose-dependently decreased the product formation of both isoforms (Table 1). Resveratrol was the most potent compound tested and the IC_50_ values of 0.49 μM (COX–1) and 0.43 μM (COX–2) were comparable for both isoforms. For EGCG, ε-viniferin and hopeaphenol the IC_50_ values to inhibit the COX–2 isoform were higher in comparison to those required to inhibit COX–1, e.g. 1.6 μM (COX–1) and11 μM (COX–2) for ε-viniferin (Table 1).

**Table 1:** Effect of polyphenols on COX activity in the different *in vitro* test systems.

	COX–1 enzyme assay	COX–2 enzyme assay	HCA-7cell line		primary monocytes	
	IC_50_ (μM) *(95% CI)	IC_50_ (μM) *(95% CI)	IC_50_ (μM) *(95% CI)	COX-2Expression ^†^	IC_50_ (μM) *(95% CI)	COX-2Expression ^†^
**Nobiletin**	no effect (up to 100 μM)	no effect (up to 100 μM)	inhibition < 50%(up to 100 μM)	100 μM:COX–2 ↓	**24**(18–34)	no effect(up to 100 μM)
**Naringenin**	no effect (up to 100 μM)	no effect (up to 100 μM)	100 μM:53% inhibition	100 μM:COX–2 ↓	**29**(27–30)	100, 30 μM:COX–2 ↓
**Genistein**	no effect (up to 30 μM)	no effect (up to 30 μM)	100 μM:54% inhibition	100 μM:COX–2 ↓	**7.4**(4.4–13)	n.d.
**Apigenin**	inhibition < 50%(up to 100 μM)	no effect (up to 100 μM)	10 μM:59% inhibition	no effect(up to 10 μM)	**2.6**(2.4–3.0)	10, 3 μM:COX–2 ↓
**EGCG**	**17**(2.6–108)	**32**(16–64)	no effect (up to 10 μM)	no effect(up to 10 μM)	no effect(up to 10 μM)	10 μM:COX–2 ↓
**Wogonin**	no effect (up to 100 μM)	no effect (up to 100 μM)	inhibition < 50%(up to 10 μM)	no effect(up to 10 μM)	**1.5**(0.89–2.5)	n.d.
**Resveratrol**	**0.49** (0.34–0.71)	**0.43** (0.27–0.67)	**4.7** (2.9–7.7)	no effect(up to 50 μM)	**2.8** (2.2–3.5)	50, 10 μM: COX–2 ↓
**ε-Viniferin**	**1.6** (0.97–2.6)	**11** (2.5–44)	no effect (up to 1 μM)	no effect (up to 1 μM)	inhibition < 50% (up to 1 μM)	no effect (up to 1 μM)
**Hopeaphenol**	**4.0** (2.4–6.7)	**22** (6.9–68)	no effect (up to 1 μM)	no effect (up to 1 μM)	inhibition < 50% (up to 1 μM)	1 μM: COX–2 ↓

n. d. no data

* IC_50_ values were calculated based on the PGE_2_ (n = 3).

^†^ COX–2 protein levels were analyzed by COX–2 specific Western Blot; decreased COX–2 protein in comparison to the control is indicated by ↓.

### Modulation of COX–2 activity in cells

**Fig 1:**
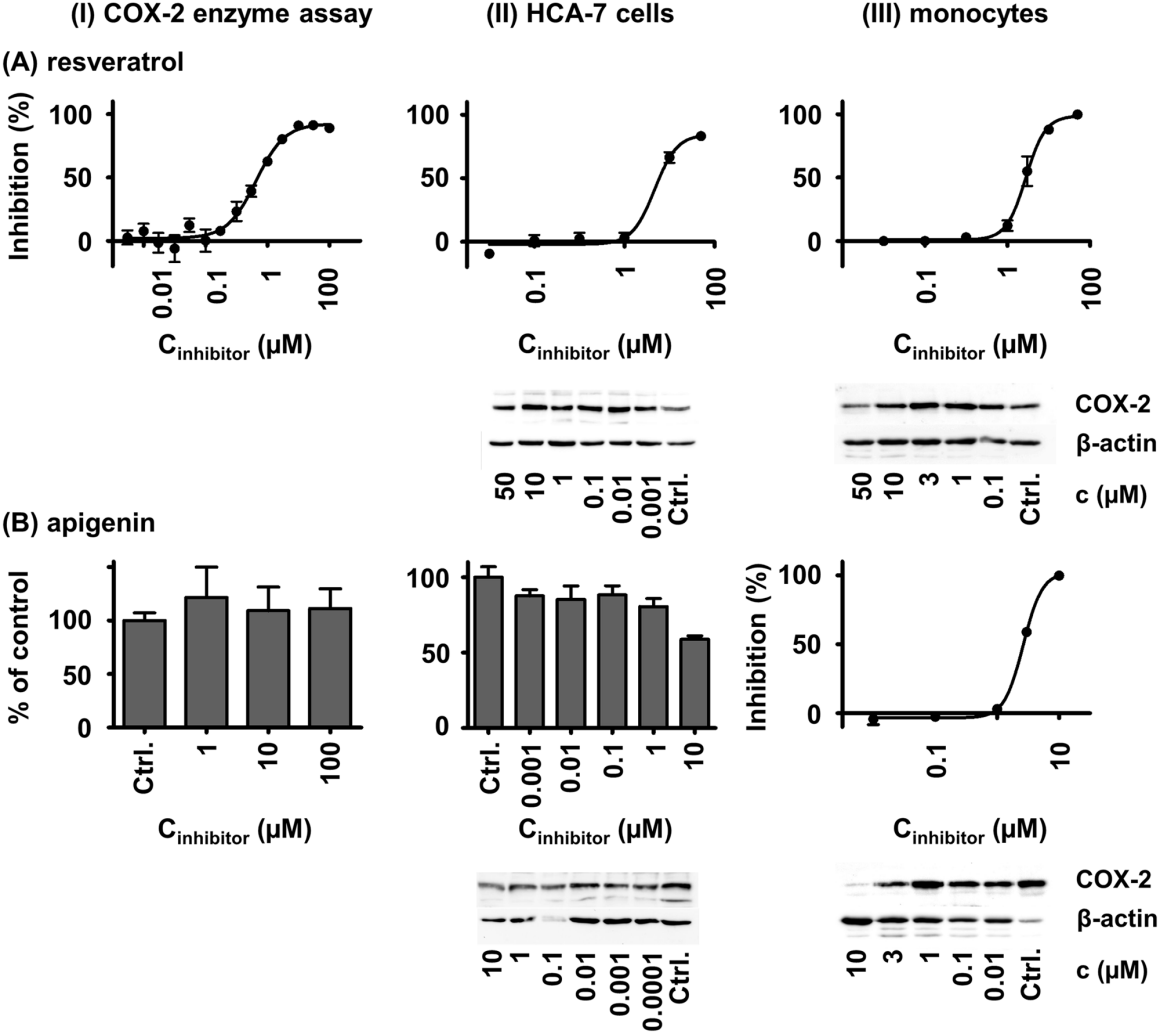
Effects of resveratrol (A) and apigenin (B) on PGE_2_ formation and COX–2 expression in the COX–2 enzyme assay (I), in HCA–7 cells (II) and in LPS-stimulated primary human monocytes (III). The inhibition (% of control) was calculated based on the PGE_2_ formation. Shown are mean ± SD.

Incubation of HCA–7 cells with non-cytotoxic concentrations of the polyphenols nobiletin, EGCG, wogonin, ε-viniferin and hopeaphenol resulted in no or only slight (inhibition < 50%) changes of PGE_2_ formation (Table 1). Naringenin (100 μM), genistein (100 μM) and apigenin (10 μM) inhibited the formation of PGE_2_, and the IC_50_ values were estimated to be about 100 μM. In contrast, resveratrol potently reduced PGE_2_ formation in HCA–7 cells at an IC_50_ of 4.7 μM (Table 1, Fig 1). A COX–2 specific Western Blot analysis of the cells treated with the polyphenols indicated for nobiletin, naringenin and genistein a decreased COX–2 expression, while all other compounds did not affect the COX–2 protein levels (Table 1).

COX–2 activity in LPS stimulated primary human monocytes was slightly reduced by ε-viniferin and hopeaphenol (inhibition < 50%, Table 1). All other polyphenols inhibited PGE_2_ formation with IC_50_ values between 1.5 and 29 μM (Table 1). The most potent polyphenols tested were wogonin (1.5 μM), apigenin (2.6 μM), resveratrol (2.8 μM) and genistein (7.4 μM). Decreased COX–2 protein levels were found in incubations with naringenin, apigenin, EGCG, resveratrol and hopeaphenol (Table 1, Fig 1).

### *In vivo* model

**Fig 2:**
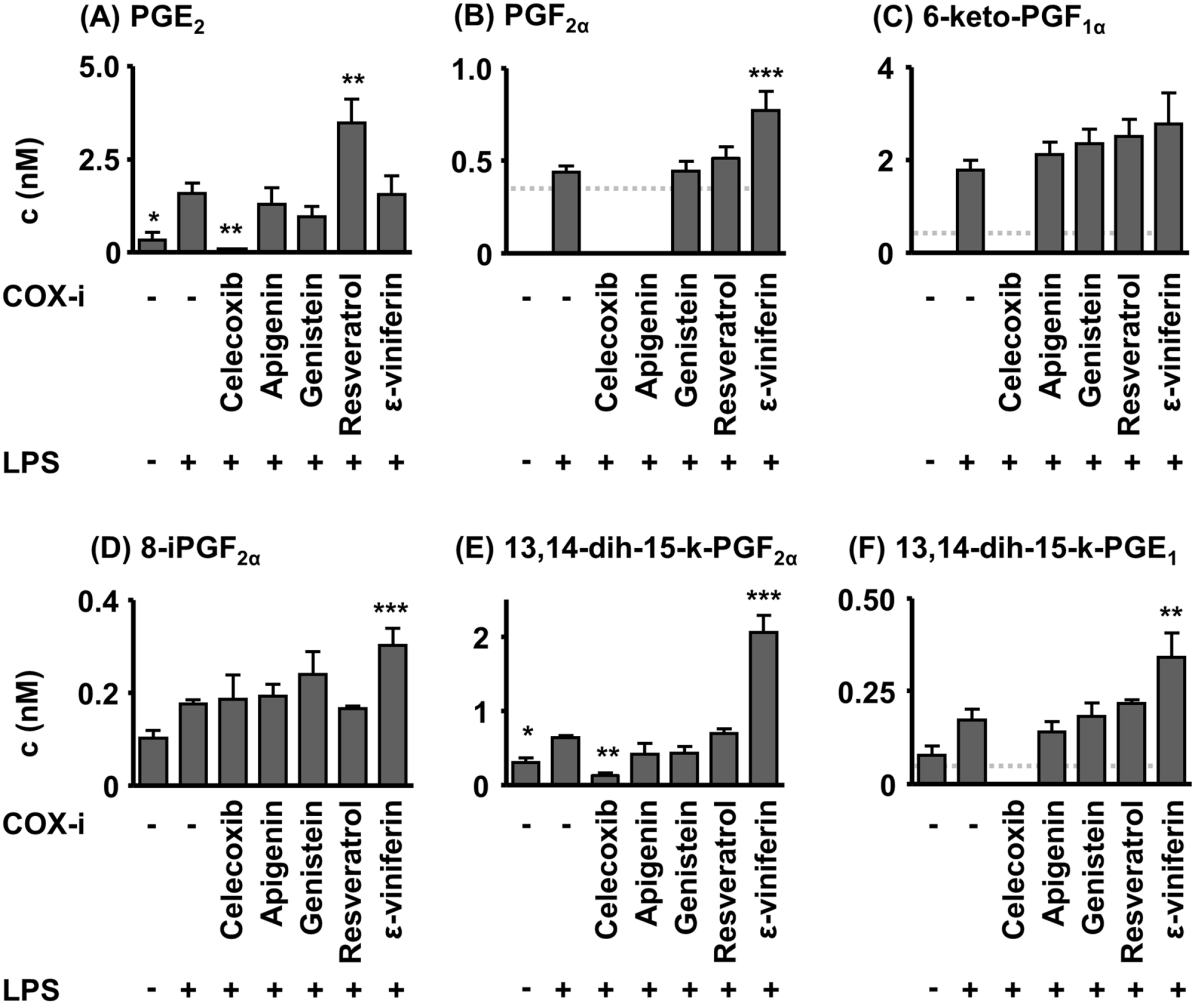
Plasma PG levels in acute (24 h) LPS induced sepsis in mice. The test compounds were administered 2 h prior to the induction of sepsis by LPS (100 mg/kg bw, i. p.). PG concentration is below the limit of quantification (LOQ, dotted line) if no bar is displayed. Shown are mean ± SEM. (n = 4–8, ANOVA followed by Dunnett‘s test *p<0.05, **p<0.01, ***p<0.001).

In acute systemic inflammation caused by LPS (i. p. 10 mg/kg, no COX-i) plasma PG levels were massively elevated compared to the control (Fig 2). The plasma concentration of the non-enzymatically formed 8-iPF_2α_ was not significantly elevated by LPS induced sepsis. Treatment with a high dose of the selective COX-2i celecoxib—serving as positive control—attenuated LPS induced PG increase: 6-keto-PGF_1α_, PGF_2α_ and 13,14-dihydro-15-keto-PGE_1_ were below the limit of quantification (LOQ). PGE_2_ (p< 0.01) and 13,14-dihydro-15-keto-PGF_2α_ (p< 0.01) were massively decreased in comparison to the LPS group (Fig 2). Treatment with the polyphenols (i. p. 100 mg/kg) did not result in decreased PG levels in comparison to the LPS group (Fig 2). The PGE_2_ levels were even elevated (p< 0.01) following resveratrol administration. For the polyphenol ε-viniferin, high levels of PGF_2α_ (p< 0.001), 13,14-dihydro-15-keto-PGF_2α_ (p< 0.001), 13,14-dihydro-15-keto-PGE_1_ (p< 0.01) and the non-enzymatically formed autoxidation marker 8-iPF_2α_ (p< 0.001) were detected.

**Fig 3:**
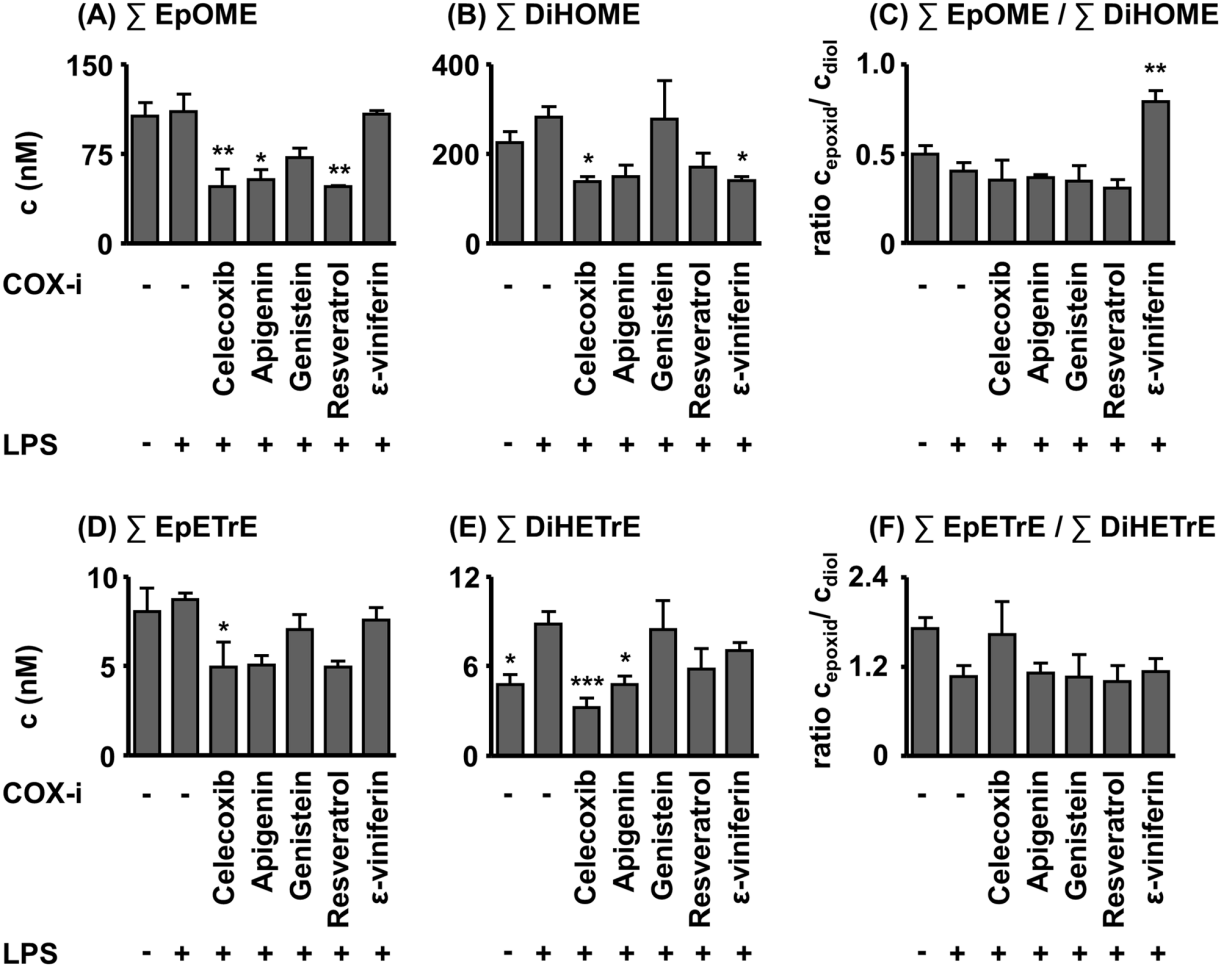
Sum of LA and AA plasma epoxy fatty acid and dihydroxy fatty acid levels in acute (24 h) LPS-induced sepsis in mice. The test compounds were administered 2 h prior induction of sepsis LPS (100 mg/kg BW, i. p.). Shown are mean ± SEM. (n = 4–8, ANOVA followed by Dunnett‘s test *p<0.05, **p<0.01, ***p<0.001).

It is noteworthy, that the COX blockade by the celecoxib group did not shunt oxylipin formation towards the CYP- or LOX pathway of the AA cascade (Fig 3, S2 Table), as described in earlier studies [20]. The epoxy- and dihydroxy-fatty acid (FA) metabolites were even decreased in the CYP pathway (Fig 3, S2 Table). A similar decrease was observed for the sum of linoleic acid (LA) derived epoxy-metabolites in the apigenin (p< 0.05) and the resveratrol (p< 0.01) group and the sum of DiHETrEs in the case of apigenin (p< 0.05) as exemplary shown in Fig 3 for the metabolites of LA and AA.

**Fig 4:**
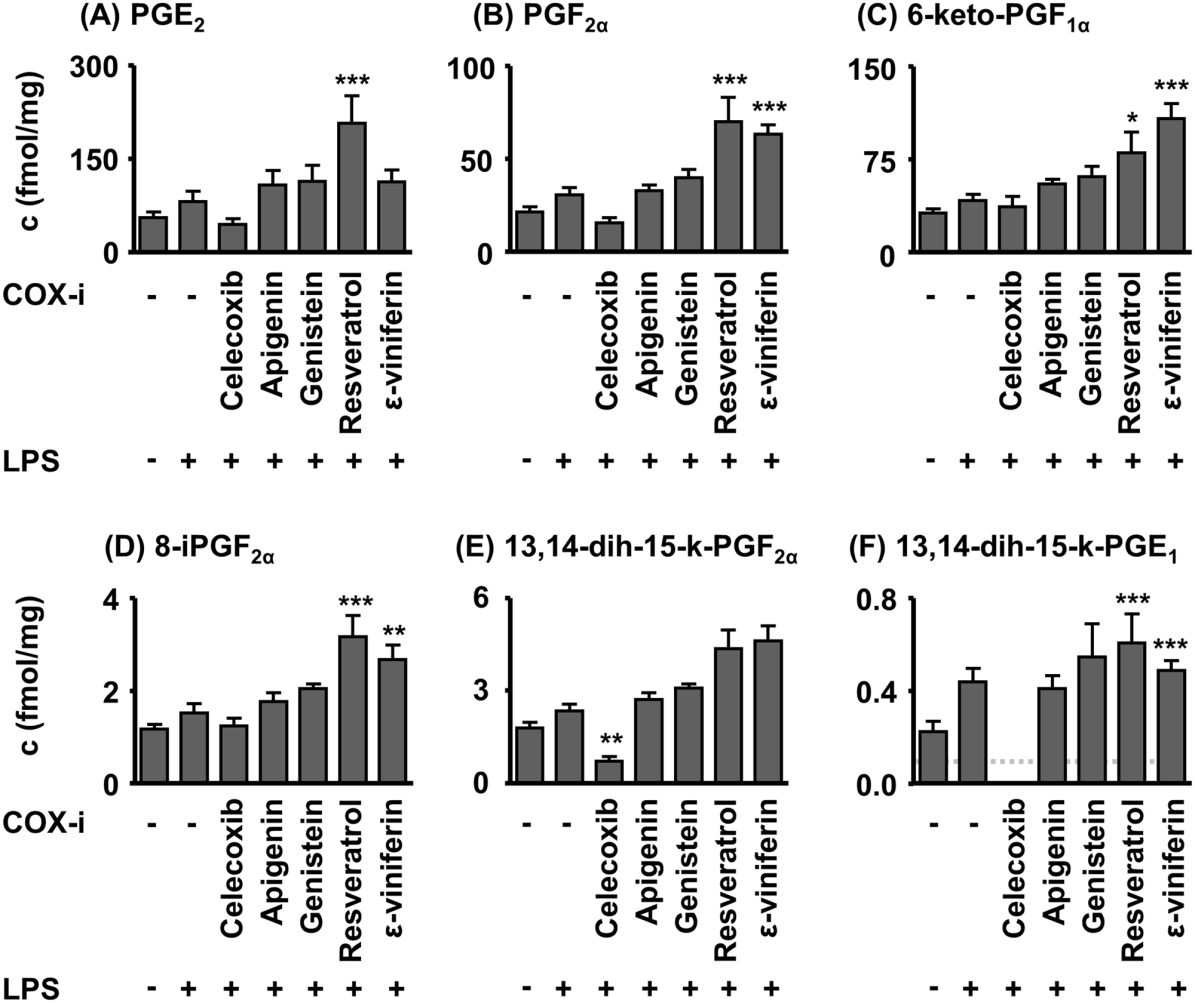
Kidney PG levels in acute (24 h) LPS induced sepsis in mice. The test compounds were administered 2 h prior induction of sepsis by LPS (100 mg/kg BW, i. p.). Shown are mean ± SEM. (n = 4–8, ANOVA followed by Dunnett‘s test *p<0.05, **p<0.01, ***p<0.001).

The kidney PG levels 24 h after LPS injection showed a similar trend as in plasma. Slightly increased PG levels were observed in the LPS group when compared to the control group (Fig 4, S3 Table). The selective COX–2 inhibitor celecoxib lowered PG concentrations in comparison to the LPS group (PGE_1_ metabolite 13,14-dihydro-15-keto-PGE_2α_ (p< 0.01), PGE_1_ metabolite 13,14-dihydro-15-keto-PGE_1_ <LOQ.). Kidney levels of PGE_2_ (p< 0.001), PGF_2α_ (p< 0.001), 6-keto-PGF_1α_ (p< 0.05), 13,14-dihydro-15-keto-PGE_1_ (p< 0.001) and 8-iPGF_2α_ (p< 0.001) in the resveratrol group were increased in comparison to the animals only receiving LPS. 6-keto-PGF1_α_ (p< 0.001), 13,14-dihydro-15-keto-PGE_1_ (p< 0.001) and 8-iPGF_2α_ (p< 0.01) kidney levels were elevated in the ε-viniferin treated mice (Fig 4).

**Fig 5:**
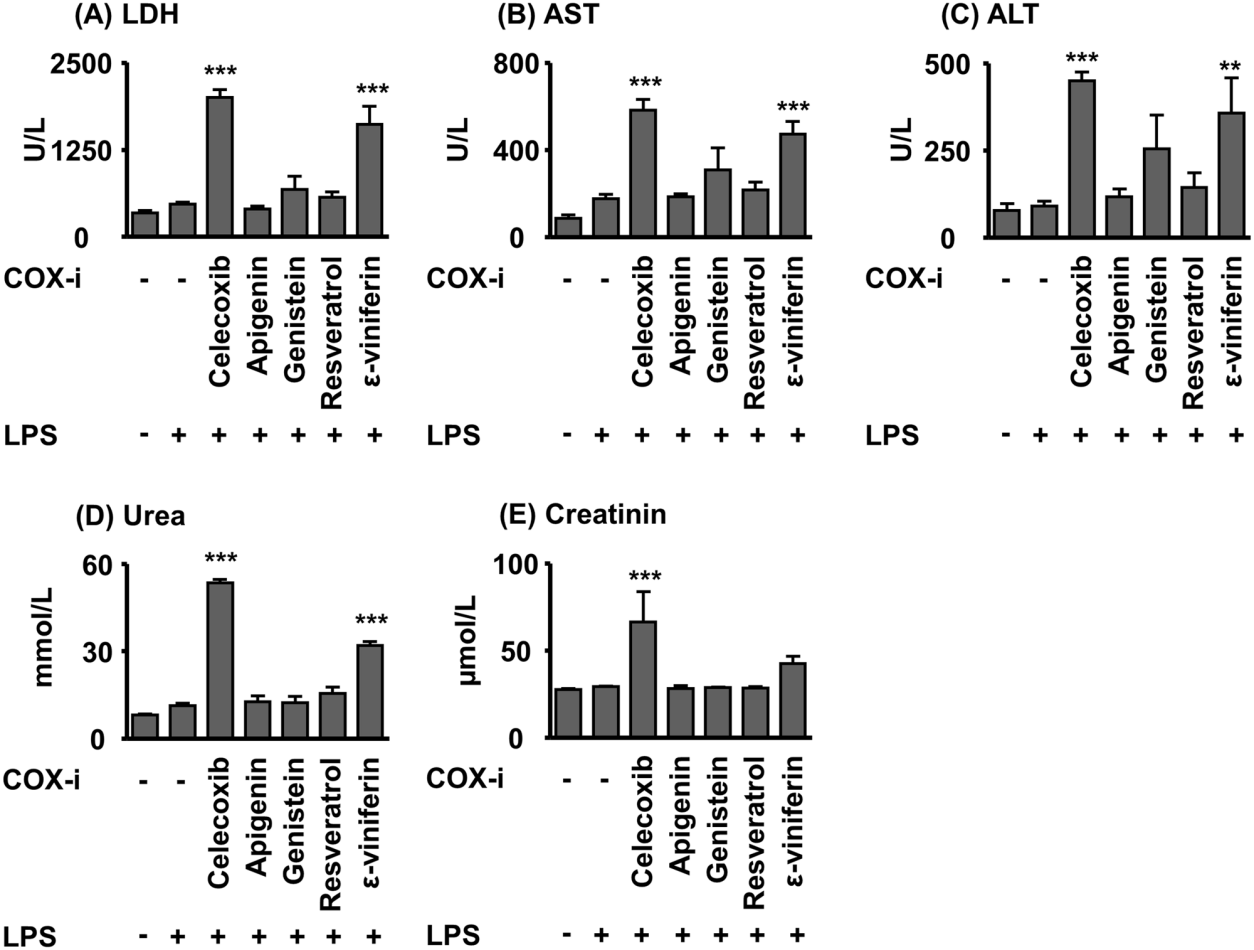
Plasma urea, and creatinine levels as well as plasma AST, ALT and LDH activities in acute (24 h) LPS-induced sepsis in mice. The test compounds were administered 2 h prior to the induction of sepsis ba LPS (100 mg/kg bw, i. p.). Shown are mean ± SEM. (n = 4–8, ANOVA followed by Dunnett‘s test *p<0.05, **p<0.01, ***p<0.001).

Clinical chemistry markers of kidney function (plasma creatinine and urea levels) were moderately increased in the LPS group, while treatment with celecoxib and ε-viniferin caused an elevation of both parameters (Fig 5) indicating worsening of renal function.

**Fig 6:**
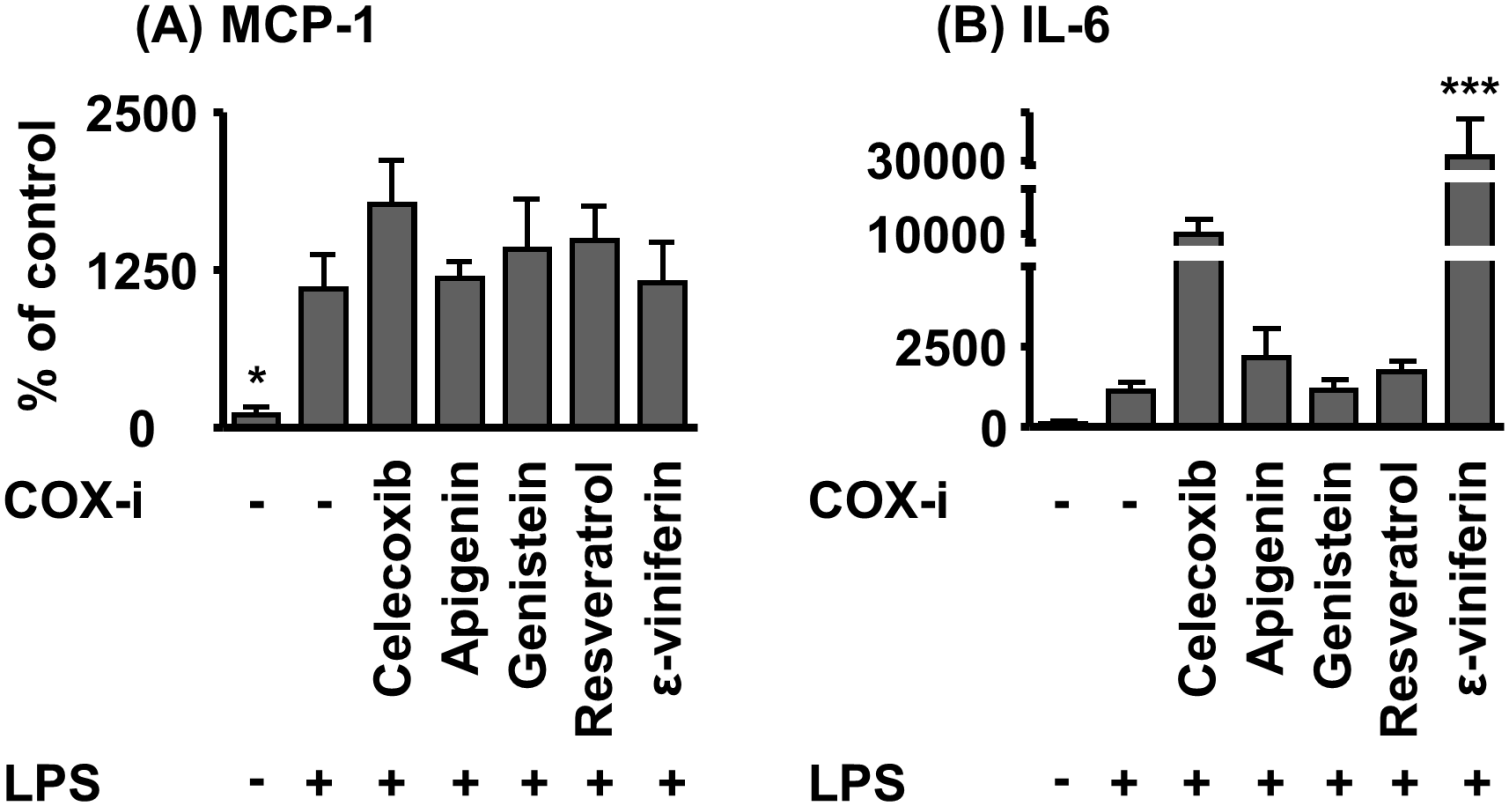
Kidney MCP–1 and IL–6 mRNA levels in acute (24 h) LPS-induced sepsis in mice. The test compounds were administered 2 h prior to the induction of sepsis by LPS (100 mg/kg bw, i. p.). Shown are mean ± SEM. (n = 3–5, ANOVA followed by Dunnett‘s test *p<0.05, **p<0.01, ***p<0.001).

The chemoattractant and pro-inflammatory molecule MCP–1 was measured by qPCR in renal tissue and revealed elevated mRNA levels in the LPS group (p <0.05 LPS versus vehicle group). Combined treatment with LPS and celecoxib or polyphenols did not affect MCP–1 levels in comparison to the LPS group (Fig 6). The pro-inflammatory IL–6 mRNA expression in the kidney was about 10-fold increased due to LPS injection in comparison to vehicle control (p > 0.05). Treatment with apigenin, resveratrol and genistein resulted in similar IL–6 mRNA elevation as in the LPS group, whereas celecoxib und ε-viniferin caused further increase IL–6 mRNA expression compared to the LPS group (celecoxib 9-fold, ε-viniferin 28-fold in comparison to LPS group, Fig 6).

In the liver tissue no change in PG levels was observed between the LPS and the control group (S1 Fig, S4 Table). Consistently, only slight increase in plasma ALT activity and a moderate increase in AST activity were observed (Fig 5). The mice treated with celecoxib, ε-viniferin and genistein showed elevated AST and ALT activity in plasma indicating aggravated liver injury of the high dose (Fig 5).

## Discussion

The current concept is that several food ingredients elicit effects on human health by a modulation of the activity of enzymes of the AA cascade [21]. Particularly for food polyphenols a large number of studies report a potentially beneficial reduction of COX–2 activity [3]. The aim of the present work was to reevaluate these findings, analyzing a library of polyphenols—which have been potent in three *in vitro* test systems—in a robust *in vivo* inflammation model for their effects on COX–2.

EGCG, resveratrol, ε-viniferin and hopeaphenol inhibited COX–1 and COX–2 in the cell free assay (Table 1). The most potent polyphenol tested was resveratrol with an IC_50_ of 0.49 μM (COX–1) and 0.43 μM (COX–2) (Table 1). The potency is consistent with previous studies reporting of IC_50_ values for resveratrol ranging from 0.5 to 0.9 μM in the case of COX–1 and from 1.0 to 3.1 in the case of COX–2 [16, 22, 23]. Thus, our data support the hypothesis that polyphenols could indeed act as COXi. It is remarkable that the polyphenols inhibited COX–2 at potency levels comparable to those of the NSAID indomethacin (0.36 μM) and selective COX-2i celecoxib (0.24 μM) in this test system (S5 Table). Both compounds are frequently used in the clinic for analgetic treatment of patients [24]. Taking the tyrosyl radical involved in the enzyme catalysis and the radical scavenging properties of polyphenols into account, the inhibition of COX by polyphenols is discussed as being a rather unspecific mechanism [25, 26]. However, the polyphenols naringenin, genistein and apigenin with a strong antioxidative capacity [27, 28] showed no effect on COX. Hence, the inhibition by EGCG, resveratrol, ε-viniferin and hopeaphenol seems not to be based on a fully unspecific mechanism.

In order to evaluate the biological relevance of enzyme inhibitors, cell assays are more predictive because the enzyme acts in its intact cellular compartment (ER) and changes at the expression level can be monitored. Moreover, the uptake of the inhibitor into the cell is taken into account. When studying biological questions in cellular systems, it is crucial to choose the most adequate cell line. COX–2 expression is increased in many types of cancer, e. g. colon, breast or lung cancer, and, thus, some cell lines derived thereof, such as the colon adenocarcinoma derived cell line HCA–7, abundantly express COX–2 [29]. Therefore, these HCA–7 cells were selected for the *in vitro* studies of the effects of polyphenols on COX–2 activity.

In HCA–7 cells, only naringenin, nobiletin, genistein, apigenin and resveratrol affected COX–2 dependent PGE_2_ formation (Table 1). In the case of resveratrol, the unchanged COX–2 protein levels after 24 hours suggest that resveratrol acts by inhibiting COX–2 activity, which is consistent with earlier findings [17]. However, at longer incubation times (48 h and 96 h) resveratrol has been shown to decrease COX–2 expression [17]. The slight change in PGE_2_ formation and the unchanged COX–2 protein levels after an incubation with apigenin are comparable to a previous study [6]. Interestingly, the effects of genistein, nobiletin and naringenin can be in part explained by a decreased COX–2 expression (Table 1). This mode of action is supported by the lack of effect on the COX–2 activity in the cell free enzyme assay. However, the effects only occurred at high concentrations of the compounds. Among the compounds tested only resveratrol effectively decreased the PGE_2_ formation in HCA–7 cells. However, its IC_50_ of 4.7 μM was more than 10-fold higher than that of celecoxib (IC_50_ 0.29 μM) and indomethacin (IC_50_ 0.58 μM) (S5 Table). Resveratrol can be rapidly glucuronidated by intestinal cells and the resulting low intracellular concentration may explain the low efficacy of resveratrol [30]. Based on these data, it seems unlikely that polyphenols could affect COX–2 expression and PGE_2_ formation of cancer cells *in vivo*.

An up-regulated expression of COX–2 in monocytes, macrophages and other cells plays a key role in acute inflammation [31]. This makes COX–2 to one of the main drug targets in inflammation. Although cell line models, e. g. the murine macrophage cell line RAW 264.7, are often used to study effects on COX–2 during inflammatory processes *in vitro* [7, 8, 12, 14], LPS stimulated primary human monocytes are closer to the *in vivo* situation in patients. In stimulated primary human monocytes all tested polyphenol potently inhibited LPS induced PGE_2_ production except for EGCG, ε-viniferin and hopeaphenol. Resveratrol, apigenin, genistein and wogonin were the most potent polyphenols with IC_50_ values below 10 μM. Although comparison of different cellular systems has its limitations, these results are consistent with earlier studies in RAW 264.7 cells reporting an IC_50_ value of 8 μM and reduced COX–2 levels for apigenin, reduced PGE_2_ formation and COX–2 levels for genistein as well as a lack of effect of EGCG on PGE_2_ levels with even a slight increase in COX–2 protein levels [10]. Incubation of LPS stimulated murine macrophages with wogonin resulted in a suppression of COX–2 protein expression and an IC_50_ of 0.3 μM [8], whereas for naringenin and nobiletin decreased COX–2 protein levels were found in LPS stimulated RAW 264.7 cells [7, 9]. The decreased COX–2 protein levels for naringenin and apigenin in combination with the absence of inhibitory effects in the enzyme assay suggest that these polyphenols reduce PGE_2_ production in primary monocytes by down regulation of COX–2 expression. As described earlier [11], resveratrol acts by two different mechanisms, the down-regulation of COX–2 protein and the inhibition of the COX–2 activity (Table 1, Fig 1). One mechanism how polyphenols could reduce COX–2 mRNA expression is modulation of nuclear factor kappa B (NF-κB) signaling [32, 33]. Part of this action may be explained by their antioxidative properties which lead to reduced oxidative endoplasmic reticulum (ER) stress and reduced NF-κB activation [34, 35].

In comparison with pharmaceutically used COX inhibitors, the polyphenols are about 100 to 1000-fold less potent in the monocyte assay with IC_50_ values of 14, 10 and 1.6 nM for celecoxib, indomethacin and dexamethasone (S5 Table).

Following consumption of polyphenol rich foods significant plasma concentrations were detected in humans [36]. Consumption of a black tea containing 0.1 g mg of EGCG resulted in maximal plasma concentrations of 0.13–0.33 μM [37]. The ingestion of grapefruit juice (0.2 g total naringenin) resulted in a plasma peak level of 6 μM total naringenin (free and conjugated form) [38]. After consumption of a single soy meal containing 3.6 μmol total genistein/kg bw, maximal plasma concentrations of 4 μM (free and conjugated form) were observed [26]. Though these concentrations are in the range of the determined IC_50_ values for COX–2 inhibition in the different assay systems (Table 1), it should be noted that the major portion of polyphenols in plasma is detected in form of its phase II metabolites, dominantly *O*-glucuronides. For example even a high single oral dose of 5 g resveratrol led to a plasma concentration of 2.3 μM, while the concentration of its phase II metabolites was several fold higher [39].

Nevertheless, these studies indicate that plasma concentrations close to their IC_50_ in the μM range could result from the oral intake of a high oral dose of polyphenols [36]. In order to evaluate potential health effects of polyphenols on COX–2 a robust *in vivo* model with a highly induced expression of COX–2 and elevated PGE_2_ levels was selected. The LPS induced sepsis model is a reliable and often used animal model to study the effects of compounds on the AA cascade and in this context in particular on the COX–2 mediated branch [40, 41]. However, this approach does not intend to utilize polyphenols to treat sepsis. Compounds were administered i. p. to prevent low intestinal absorption and tested in a high dose to ensure that even low to moderate inhibitory effects can be detected. Similar high doses have been used in previous studies investigating the effect of apigenin and resveratrol or celecoxib on LPS induced sepsis [20, 33, 42]. It should be noted that celecoxib and ε-viniferin caused moderate elevation of liver function parameters AST, ALT, and LDH indicating liver function impairment (Fig 5). As previously described, celecoxib reduced plasma and kidney PG levels significantly [20], thus demonstrating that the model allows to detect modulation of COX–2 *in vivo*. Therefore, celecoxib was a suitable positive control in the LPS induced sepsis model. None of the polyphenols tested attenuated the PG levels. Taking the high dose administered into account, this observation suggests that the potential to affect COX–2 *in vivo* is negligible. Resveratrol as well as its dimer increased the PGE_2_ plasma concentration, which indicate that the stilbene might even aggravate at high concentrations the inflammatory process (Fig 2).

The targeted metabolomics approach enables the parallel detection of a large number of products being part of the COX pathway. This allowed us to characterize the COX–2 inhibition in more detail. In animals treated with celecoxib not only PGE_2_ but also the plasma metabolites of PGI_2_ (6-keto-PGF_1α_), PGE_1_ (13,14-dihydro-15-keto-PGE_1_) and PGF_2α_ (13,14-dihydro-15-keto-PGF_2α_) were decreased, thereby additionally supporting an inhibition of COX–2 by celecoxib. However, all these metabolites were not lowered by treatment with polyphenols (Fig 2), thus substantiating the fact that the polyphenols cannot modulate COX–2 *in vivo*.

The targeted metabolomics approach led to an interesting additional finding: Both apigenin and resveratrol reduced the levels of the CYP derived epoxy-FAs and their hydrolysis products (dihydroxy-FAs, Fig 3). This indicates that CYPs are inhibited by these compounds, a mode of action of polyphenols that is well known for drug metabolizing CYPs [43]. This unexpected activity of the compounds should be addressed in further studies.

The treatment with polyphenols did not attenuate tissue inflammation of the kidney or systemic liver function and renal function impairment as measured by clinical chemistry in the LPS induced sepsis model (Figs 5 and 6). Thus, it has to be concluded that the food ingredients do not alleviate the inflammation and organ damage caused by acute sepsis under the conditions of our study. Our study is in line with Larossa et al. [42] who observed no effect on the levels of a number of inflammatory biomarkers after resveratrol treatment. Nicholas et al. studied the effect of i.p. administration of a similar dose of apigenin (50 mg/kg bw) in a high dose LPS (37.5 mg/kg bw) mouse model and reported reduced mortality at 28–42 h post LPS administration. This protective effect was discussed in context of modulation of NF- κB signaling and reduced pro-inflammatory cytokine release [33]. In our study we investigated the effect of apigenin in a milder LPS (10 mg/kg bw) model at a single time point 24 h after LPS treatment therefore we cannot conclude on mortality. However, we did not see an effect on the pro-inflammatory cytokines IL–6 and MCP–1. Though, we did not observe changes in COX–2 activity based on PG levels *in vivo* the reduced COX–2 expression in monocytes is consistent with blockade NF-κB signaling reported by Nicholas et al *in vitro*.

Overall our data support earlier findings that food polyphenols inhibit COX–2 *in vitro*. However, if more biological relevant systems are used, the efficacies of these natural products are lower in comparison to drugs (factor 10 in cancer cells, factor 100–1000 in primary monocytes). *In vivo*, even a high dose of polyphenols had no effect on COX–2 activity on acute inflammation in the LPS sepsis model. Taking the poor bioavailability of polyphenols [44] into account, it seems highly unlikely that the highest dose that can possible be ingested would modulate acute inflammation. However, direct effects in the gastrointestinal tract might take place, as reduced COX–2 expression and PGE_2_ production have been observed for resveratrol in different murine colitis models [45, 46].

## Supporting Information

S1 Fig Liver PG levels in acute (24 h) LPS induced sepsis in mice. The test compounds were administered 2 h prior induction of sepsis by LPS (100 mg/kg BW, i. p.). Shown are mean ± SEM. (n = 4–8, ANOVA followed by Dunnett‘s test *p<0.05, **p<0.01, ***p<0.001).
(DOCX)

S1 Table Parameters of the LC-MS method used for the quantification of oxylipins in the *in vivo* model. Shown are the analytes with their mass transitions for quantification in the scheduled SRM mode, electronic MS parameters (declustering potential (DP), collision energy (CE), collision exit potential (CXP)), retention time and the calibration range (lower limit of quantification (LLOQ), upper limit of quantification (ULOQ)).
(DOCX)

S2 Table Oxylipin concentrations in plasma 24 h after LPS treatment. (DOCX)

S3 Table Oxylipin concentrations in kidney tissue 24 h after LPS treatment. (DOCX)

S4 Table Oxylipin concentrations in liver tissue 24 h after LPS treatment. (DOCX)

S5 Table Effect of commonly used anti-inflammatory drugs on COX activity under the same assay conditions [18]. (DOCX)

S6 Table Intensity of COX–2 bands shown in the western blots in Fig 1. The ratio of the intensity of the COX–2 band and β-actin band are shown as % of control.
(DOCX)

## Abbreviations

AA: arachidonic acid
ACN: acetonitrile
ALT: alanine transaminase
AST: aspartate transaminase
COX: cyclooxygenase
CYP: cytochrome P450 monooxygenase
DiHDPE: dihydroxy docosopentaenoic acid
DiHETE: dihydroxy eicosatetraenoic acid
DiHETrE: dihydroxy eicosatrienoic acid
DiHODE: dihydroxy octadecadienoic acid
DiHOME: dihydroxy octadecenoic acid
DMEM: Dulbecco’s Modified Eagle Medium
DMSO: dimethyl sulfoxide
EKODE: epoxy-keto-octadecenoic acid
EpDPE: epoxy docosapentaenoic acid
EpETE: epoxy eicosatetraenoic acid
EpETrE: epoxy eicosatrienoic acid
EpODE: epoxy octadecadienoic acid
EpOME: epoxy octadecenoic acid
FA: fatty acid
HDHA: hydroxy docosahexaenoic acid
HEPE: hydroxy eicosapentaenoic acid
HETE: hydroxy eicosatetraenoic acid
HETrE: hydroxyl eicosatrienoic acid
HODE: hydroxy octadecadienoic acid
HOTrE: hydroxy octadecatrienoic acid
IL: interleukin
iP: isoprostane
LC: liquid chromatography
LLOQ: lower limit of quantification
LOQ: limit of quantification
LOX: lipoxygenase
LPS: lipopolysaccharide
LT: leukotriene
m/z: mass to charge ratio
MS: mass spectrometry
NSAID: non-steroidal anti-inflammatory drug
PG: prostaglandin
RPMI: Roswell Park Memorial Institute medium
SD: standard deviation
SEM: standard error mean
SPE: solid phase extraction
TRIS: tris(hydroxymethyl)aminomethane
Tx: thromboxane
ULOQ: Upper limit of quantification

## References

[1] Arts ICW, Hollman PCH. Polyphenols and disease risk in epidemiologic studies. Am J Clin Nutr. 2005;81(1):317S–25S. 15640497 10.1093/ajcn/81.1.317S

[2] Riboli E, Norat T. Epidemiologic evidence of the protective effect of fruit and vegetables on cancer risk. Am J Clin Nutr. 2003;78(3):559S–69S. 12936950 10.1093/ajcn/78.3.559S

[3] Garcia-Lafuente A, Guillamon E, Villares A, Rostagno MA, Martinez JA. Flavonoids as anti-inflammatory agents: implications in cancer and cardiovascular disease. Inflamm Res. 2009;58(9):537–52. 10.1007/s00011-009-0037-3 19381780

[4] Murakami A, Ohigashi H. Targeting NOX, INOS and COX–2 in inflammatory cells: Chemoprevention using food phytochemicals. Int J Cancer. 2007;121(11):2357–63. 17893865 10.1002/ijc.23161

[5] Buczynski MW, Dumlao DS, Dennis EA. Thematic Review Series: Proteomics. An integrated omics analysis of eicosanoid biology. J Lipid Res. 2009;50(6):1015–38. 10.1194/jlr.R900004-JLR200 19244215 PMC2681385

[6] Al-Fayez M, Cai H, Tunstall R, Steward W, Gescher A. Differential modulation of cyclooxygenase-mediated prostaglandin production by the putative cancer chemopreventive flavonoids tricin, apigenin and quercetin. Cancer Chemoth Pharm. 2006;58(6):816–25. 10.1007/s00280-006-0228-3 16552572

[7] Chao C-L, Weng C-S, Chang N-C, Lin J-S, Kao S-T, Ho F-M. Naringenin more effectively inhibits inducible nitric oxide synthase and cyclooxygenase–2 expression in macrophages than in microglia. Nutr Res. 2010;30(12):858–64. 10.1016/j.nutres.2010.10.011 21147369

[8] Chi YS, Jong HG, Son KH, Chang HW, Kang SS, Kim HP. Effects of naturally occurring prenylated flavonoids on enzymes metabolizing arachidonic acid: Cyclooxygenases and lipoxygenases. Biochem Pharmacol. 2001;62(9):1185–91. 11705451 10.1016/s0006-2952(01)00773-0

[9] Choi S-Y, Hwang J-H, Ko H-C, Park J-G, Kim S-J. Nobiletin from citrus fruit peel inhibits the DNA-binding activity of NF-kB and ROS production in LPS-activated RAW 264.7 cells. J Ethnopharmacol. 2007;113(1):149–55. 17611060 10.1016/j.jep.2007.05.021

[10] Liang Y-C, Huang Y-T, Tsai S-H, Lin-Shiau S-Y, Chen C-F, Lin J-K. Suppression of inducible cyclooxygenase and inducible nitric oxide synthase by apigenin and related flavonoids in mouse macrophages. Carcinogenesis. 1999;20(10):1945–52. 10.1093/carcin/20.10.1945 10506109

[11] Subbaramaiah K, Chung WJ, Michaluart P, Telang N, Tanabe T, Inoue H, et al Resveratrol Inhibits Cyclooxygenase–2 Transcription and Activity in Phorbol Ester-treated Human Mammary Epithelial Cells. J Biol Chem. 1998;273(34):21875–82. 10.1074/jbc.273.34.21875 9705326

[12] Murakami A, Nakamura Y, Torikai K, Tanaka T, Koshiba T, Koshimizu K, et al Inhibitory Effect of Citrus Nobiletin on Phorbol Ester-induced Skin Inflammation, Oxidative Stress, and Tumor Promotion in Mice. Cancer Res. 2000;60(18):5059–66. 11016629

[13] Raso GM, Meli R, Di Carlo G, Pacilio M, Di Carlo R. Inhibition of inducible nitric oxide synthase and cyclooxygenase–2 expression by flavonoids in macrophage J774A.1. Life Sci. 2001;68(8):921–31. 11213362 10.1016/s0024-3205(00)00999-1

[14] Wakabayashi I, Yasui K. Wogonin inhibits inducible prostaglandin E2 production in macrophages. Eur J Pharmacol. 2000;406(3):477–81. 11040356 10.1016/s0014-2999(00)00695-6

[15] Peng G, Dixon DA, Muga SJ, Smith TJ, Wargovich MJ. Green tea polyphenol (−)-epigallocatechin-3-gallate inhibits cyclooxygenase–2 expression in colon carcinogenesis. Mol Carcinog. 2006;45(5):309–19. 16508969 10.1002/mc.20166

[16] Cao H, Yu R, Tao Y, Nikolic D, van Breemen RB. Measurement of cyclooxygenase inhibition using liquid chromatography tandem mass spectrometry. J Pharmaceut Biomed. 2011;54(1):230–5. 10.1016/j.jpba.2010.08.001 PMC2946453 20817448

[17] Sale S, Tunstall RG, Ruparelia KC, Potter GA, Steward WP, Gescher AJ. Comparison of the effects of the chemopreventive agent resveratrol and its synthetic analog trans 3,4,5,4′-tetramethoxystilbene (DMU–212) on adenoma development in the ApcMin+ mouse and cyclooxygenase–2 in human-derived colon cancer cells. Int J Cancer. 2005;115(2):194–201. 15688382 10.1002/ijc.20884

[18] Willenberg I, Meschede AK, Schebb NH. Determining cyclooxygenase–2 activity in three different test systems utilizing online-solid phase extraction-liquid chromatography-mass spectrometry for parallel quantification of prostaglandin E2, D2 and thromboxane B2. J Chromatogr A. 2015;1391:40–8. 10.1016/j.chroma.2015.02.059 25777050

[19] Ostermann AI, Willenberg I, Schebb NH. Comparison of sample preparation methods for the quantitative analysis of eicosanoids and other oxylipins in plasma by means of LC-MS/MS. Analytical and bioanalytical chemistry. 2015;407(5):1403–14. 10.1007/s00216-014-8377-4 25542569

[20] Schmelzer KR, Inceoglu B, Kubala L, Kim I-H, Jinks SL, Eiserich JP, et al Enhancement of antinociception by coadministration of nonsteroidal anti-inflammatory drugs and soluble epoxide hydrolase inhibitors. Proc Natl Acad Sci. 2006;103(37):13646–51. 10.1073/pnas.0605908103 16950874 PMC1564210

[21] Mitjavila MT, Moreno JJ. The effects of polyphenols on oxidative stress and the arachidonic acid cascade. Implications for the prevention/treatment of high prevalence diseases. Biochem Pharmacol. 2012;84(9):1113–22. 10.1016/j.bcp.2012.07.017 22858365

[22] Kang SS, Cuendet M, Endringer DC, Croy VL, Pezzuto JM, Lipton MA. Synthesis and biological evaluation of a library of resveratrol analogues as inhibitors of COX–1, COX–2 and NF-Î°B. Bioorg Med Chem. 2009;17(3):1044–54. 10.1016/j.bmc.2008.04.031 18487053

[23] Murias M, Handler N, Erker T, Pleban K, Ecker G, Saiko P, et al Resveratrol analogues as selective cyclooxygenase–2 inhibitors: synthesis and structure activity relationship. Bioorg Med Chem. 2004;12(21):5571–8. 15465334 10.1016/j.bmc.2004.08.008

[24] Ungprasert P, Cheungpasitporn W, Crowson CS, Matteson EL. Individual non-steroidal anti-inflammatory drugs and risk of acute kidney injury: A systematic review and meta-analysis of observational studies. Eur J Intern Med. 2015. 10.1016/j.ejim.2015.03.008 25862494

[25] Simmons DL, Botting RM, Hla T. Cyclooxygenase Isozymes: The Biology of Prostaglandin Synthesis and Inhibition. Pharmacol Rev. 2004;56(3):387–437. 10.1124/pr.56.3.3 15317910

[26] Gryglewski RJ, Krobut R, Robak J, Swies J. On the mechanism of antithrombotic action of flavonoids. Biochem Pharmacol. 1987;36(3):317–22. 3101704 10.1016/0006-2952(87)90288-7

[27] Rice-Evans CA, Miller NJ, Bolwell PG, Bramley PM, Pridham JB. The Relative Antioxidant Activities of Plant-Derived Polyphenolic Flavonoids. Free Rad Res. 1995;22(4):375–83. 10.3109/10715769509145649 . 7633567

[28] Soobrattee MA, Neergheen VS, Luximon-Ramma A, Aruoma OI, Bahorun T. Phenolics as potential antioxidant therapeutic agents: Mechanism and actions. Mut Res. 2005;579(1–2):200–13. 16126236 10.1016/j.mrfmmm.2005.03.023

[29] Sharma RA, Gescher A, Plastaras JP, Leuratti C, Singh R, Gallacher-Horley B, et al Cyclooxygenase–2, malondialdehyde and pyrimidopurinone adducts of deoxyguanosine in human colon cells. Carcinogenesis. 2001;22(9):1557–60. 10.1093/carcin/22.9.1557 11532880

[30] Willenberg I, Brauer W, Empl MT, Schebb NH. Development of a Rapid LC-UV Method for the Investigation of Chemical and Metabolic Stability of Resveratrol Oligomers. J Agr Food Chem. 2012;60(32):7844–50. 10.1021/jf302136t 22808987

[31] Ricciotti E, Fitzgerald GA. Prostaglandins and Inflammation. Arterioscler Thromb Casc Biol. 2011;31(5):986–1000. 10.1161/atvbaha.110.207449 PMC3081099 21508345

[32] Romier B, Van De Walle J, During A, Larondelle Y, Schneider YJ. Modulation of signalling nuclear factor-kappaB activation pathway by polyphenols in human intestinal Caco–2 cells. Br J Nutr. 2008;100(3):542–51. 10.1017/S0007114508966666 . 18377686

[33] Nicholas C, Batra S, Vargo MA, Voss OH, Gavrilin MA, Wewers MD, et al Apigenin Blocks Lipopolysaccharide-Induced Lethality In Vivo and Proinflammatory Cytokines Expression by Inactivating NF-Î°B through the Suppression of p65 Phosphorylation. J Immunol. 2007;179(10):7121–7. 10.4049/jimmunol.179.10.7121 17982104

[34] Tam AB, Mercado EL, Hoffmann A, Niwa M. ER stress activates NF-kappaB by integrating functions of basal IKK activity, IRE1 and PERK. PloS one. 2012;7(10):e45078 10.1371/journal.pone.0045078 23110043 PMC3482226

[35] Tsatsanis C, Androulidaki A, Venihaki M, Margioris AN. Signalling networks regulating cyclooxygenase–2. The international journal of biochemistry & cell biology. 2006;38(10):1654–61. 10.1016/j.biocel.2006.03.021 . 16713323

[36] Manach C, Scalbert A, Morand C, Remesy C, Jimenez L. Polyphenols: food sources and bioavailability. Am J Clin Nutr. 2004;79(5):727–47. 15113710 10.1093/ajcn/79.5.727

[37] Scalbert A, Williamson G. Dietary Intake and Bioavailability of Polyphenols. Am J Clin Nutr. 2000;130(8):2073S–85S. 10.1093/jn/130.8.2073S 10917926

[38] Erlund I, Meririnne E, Alfthan G, Aro A. Plasma Kinetics and Urinary Excretion of the Flavanones Naringenin and Hesperetin in Humans after Ingestion of Orange Juice and Grapefruit Juice. J Nutr. 2001;131(2):235–41. 11160539 10.1093/jn/131.2.235

[39] Boocock DJ, Faust GES, Patel KR, Schinas AM, Brown VA, Ducharme MP, et al Phase I dose escalation pharmacokinetic study in healthy volunteers of resveratrol, a potential cancer chemopreventive agent. Cancer Epidem Biomar. 2007;16(6):1246–52. ISI:000247163100032. 10.1158/1055-9965.EPI-07-0022 17548692

[40] Reddy RC, Chen GH, Tateda K, Tsai WC, Phare SM, Mancuso P, et al Selective inhibition of COX–2 improves early survival in murine endotoxemia but not in bacterial peritonitis. Am J Physiol Lung Cell Mol Physiol. 2001;281(3):L537–L43. 11504678 10.1152/ajplung.2001.281.3.L537

[41] Schmelzer KR, Kubala L, Newman JW, Kim I-H, Eiserich JP, Hammock BD. Soluble epoxide hydrolase is a therapeutic target for acute inflammation. Proc Natl Acad Sci. 2005;102(28):9772–7. 10.1073/pnas.0503279102 15994227 PMC1168955

[42] Larrosa M, Azorin-Ortuno M, Yanez-Gascon MJ, Garcia-Conesa MT, Tomas-Barberan F, Espin JC. Lack of effect of oral administration of resveratrol in LPS-induced systemic inflammation. Eur J Nutr. 2011;50(8):673–80. 10.1007/s00394-011-0178-3 21373948

[43] Detampel P, Beck M, Krähenbül S, Huwyler J. Drug interaction potential of resveratrol. Drug Metab Rev. 2012;44(3):253–65. 10.3109/03602532.2012.700715 . 22788578

[44] Manach C, Williamson G, Morand C, Scalbert A, Rémésy C. Bioavailability and bioefficacy of polyphenols in humans. I. Review of 97 bioavailability studies. Am J Clin Nutr. 2005;81(1):230S–42S. 15640486 10.1093/ajcn/81.1.230S

[45] Martín AR, Villegas I, Sánchez-Hidalgo M, De La Lastra CA. The effects of resveratrol, a phytoalexin derived from red wines, on chronic inflammation induced in an experimentally induced colitis model. Br J Pharmacol. 2006;147(8):873–85. 16474422 10.1038/sj.bjp.0706469 PMC1760707

[46] Singh UP, Singh NP, Singh B, Hofseth LJ, Price RL, Nagarkatti M, et al Resveratrol (Trans–3,5,4'-trihydroxystilbene) Induces Silent Mating Type Information Regulation–1 and Down-Regulates Nuclear Transcription Factor-kB Activation to Abrogate Dextran Sulfate Sodium-Induced Colitis. The Journal of Pharmacology and Experimental Therapeutics. 2009;332(3):829–39. 10.1124/jpet.109.160838 19940103 PMC2835444

