# Supplementary material for: Food Polyphenols Fail to Cause a Biologically Relevant Reduction of COX-2 Activity
Source: PLoS One. 2015 Oct 6;10(10):e0139147. doi: 10.1371/journal.pone.0139147 (PMC4594923; doi:10.1371/journal.pone.0139147)
Supplement: S1 Fig — The test compounds were administered 2 h prior induction of sepsis by LPS (100 mg/kg BW, i. p.). Shown are mean ± SEM. (n = 4–8, ANOVA followed by Dunnett‘s test *p<0.05, **p<0.01, ***p<0.001). (DOCX) [file pone.0139147.s001.docx]

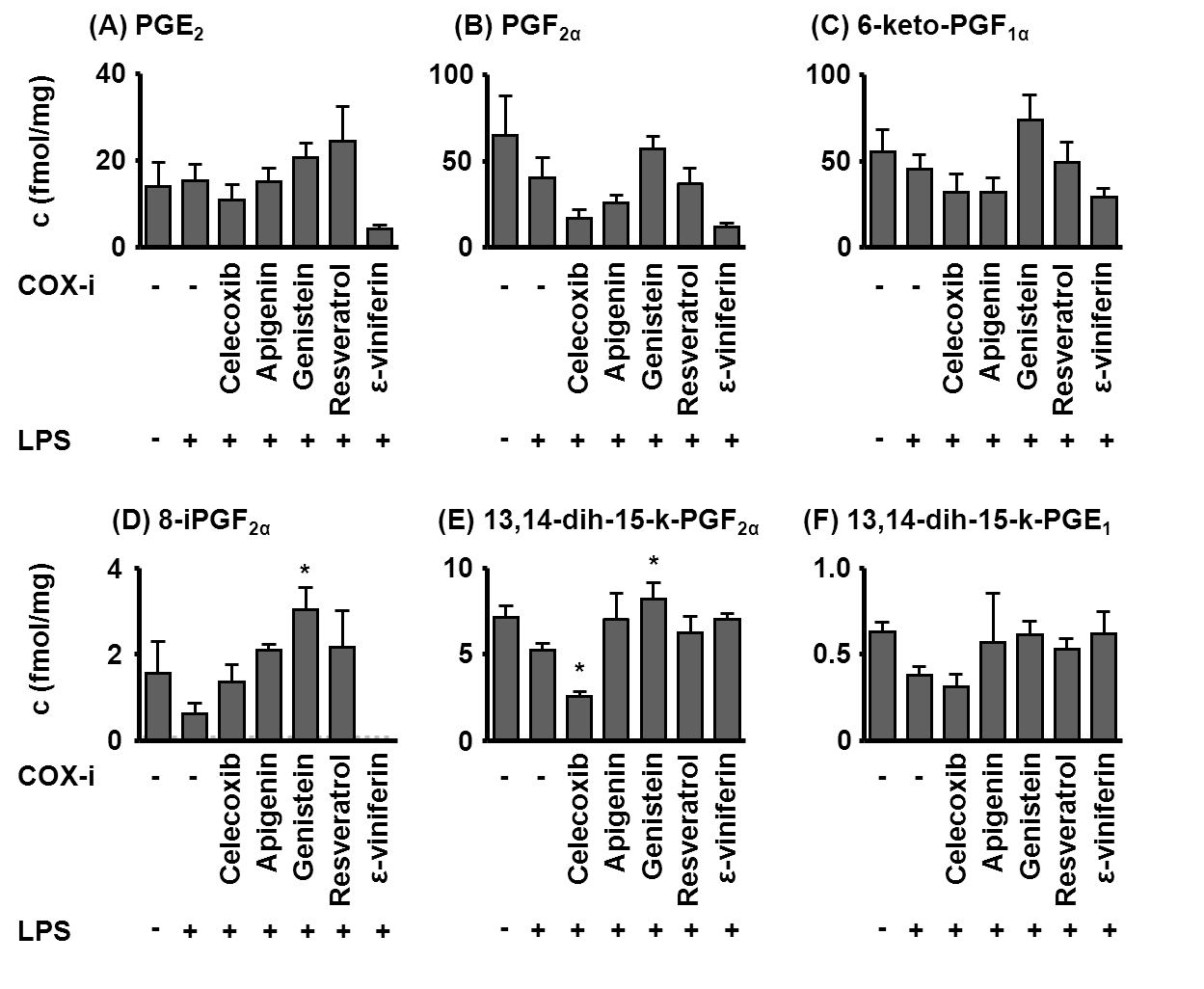


**Fig. S1:** Liver PG levels 24 h in acute (24 h) LPS-induced sepsis in mice. The test compounds were administered 2 h prior to the induction of sepsis by LPS (100 mg/kg bw, i. p.). Shown are mean ± SEM. (n= 4-8, ANOVA followed by Dunnett‘s test *p<0.05, **p<0.01, ***p<0.001).
