# Supplementary material for: Food Polyphenols Fail to Cause a Biologically Relevant Reduction of COX-2 Activity
Source: PLoS One. 2015 Oct 6;10(10):e0139147. doi: 10.1371/journal.pone.0139147 (PMC4594923; doi:10.1371/journal.pone.0139147)
Supplement: S1 Table — Shown are the analytes with their mass transitions for quantification in the scheduled SRM mode, electronic MS parameters (declustering potential (DP), collision energy (CE), collision exit potential (CXP)), retention time and the calibration range (lower limit of quantification (LLOQ), upper limit of quantification (ULOQ)). (DOCX) [file pone.0139147.s002.docx]

**Table S1:** Parameters of the LC-MS method used for the quantification of oxylipins in the *in vivo* model. Shown are the analytes with their mass transitions for quantification in the scheduled SRM mode, electronic MS parameters (declustering potential (DP), collision energy (CE), collision exit potential (CXP)), retention time and the calibration range (lower limit of quantification (LLOQ), upper limit of quantification (ULOQ)).

|  | **Mass transition** | | **MS parameter** | | | **Internal standard** | **retention time (min)** | **LLOQ ^*^ (nM)** | **ULOQ ^†^ (nM)** |
| --- | --- | --- | --- | --- | --- | --- | --- | --- | --- |
| **Analyte** | **m/z (Q1)** | **m/z (Q2)** | **DP (V)** | **CE (V)** | **CXP (V)** |  |  |  |  |
| 20-COOH-LTB_4_ | 365.2 | 347.2 | -80 | -25 | -8 | ^2^H_4_-TxB_2_ | 3.21 | 1.00 | 200 |
| 6-keto-PGF_1α_ | 369.3 | 163.2 | -70 | -36 | -6 | ^2^H_4_-6-keto-PGF_1a_ | 3.23 | 0.90 | 361 |
| Resolvin E_1_ | 349.3 | 195.0 | -65 | -22 | -10 | ^2^H_4_-TxB_2_ | 3.24 | 1.20 | 480 |
| 20-OH-LTB_4_ | 351.2 | 195.2 | -80 | -25 | -8 | ^2^H_4_-TxB_2_ | 3.36 | 0.25 | 200 |
| TxB_3_ | 367.3 | 169.3 | -70 | -34 | -8 | ^2^H_4_-TxB_2_ | 3.39 | 0.50 | 500 |
| TxB_1_ | 371.3 | 171.2 | -70 | -34 | -10 | ^2^H_4_-TxB_2_ | 3.76 | 0.50 | 500 |
| 8-i-PGF_2α_ | 353.3 | 193.3 | -100 | -32 | -4 | ^2^H_4_-PGE_2_ | 3.92 | 0.10 | 500 |
| TXB_2_ | 369.2 | 169.1 | -60 | -25 | -7 | ^2^H_4_-TxB_2_ | 3.95 | 0.63 | 500 |
| PGE_3_ | 349.3 | 269.2 | -60 | -22 | -6 | ^2^H_4_-PGE_2_ | 4.01 | 0.30 | 120 |
| 11β-PGF_2α_ | 353.3 | 193.1 | -30 | -36 | -12 | ^2^H_4_-PGE_2_ | 4.04 | 1.00 | 500 |
| 5-i-PGF_2α_ | 353.3 | 115.1 | -60 | -38 | -11 | ^2^H_4_-PGE_2_ | 4.14 | 1.00 | 500 |
| PGD_3_ | 349.3 | 269.2 | -60 | -22 | -6 | ^2^H_4_-PGE_2_ | 4.22 | 1.00 | 200 |
| 9,12,13-TriHOME | 329.2 | 211.1 | -80 | -32 | -10 | ^2^H_4_-PGE_2_ | 4.29 | 1.25 | 1000 |
| PGF_1α_ | 355.4 | 293.2 | -90 | -36 | -6 | ^2^H_4_-PGE_2_ | 4.36 | 0.10 | 500 |
| 9,10,13-TriHOME | 329.2 | 171.1 | -80 | -32 | -8 | ^2^H_4_-PGE_2_ | 4.37 | 0.50 | 400 |
| PGF_2α_ | 353.2 | 309.2 | -80 | -26 | -7 | ^2^H_4_-PGE_2_ | 4.38 | 0.70 | 281 |
| PGE_2_ | 351.2 | 271.3 | -60 | -24 | -6 | ^2^H_4_-PGE_2_ | 4.57 | 0.10 | 200 |
| PGE_1_ | 353.3 | 317.2 | -60 | -20 | -6 | ^2^H_4_-PGE_2_ | 4.70 | 0.33 | 260 |
| PGD_1_ | 353.3 | 317.2 | -60 | -20 | -6 | ^2^H_4_-PGD_2_ | 4.81 | 0.50 | 200 |
| PGD_2_ | 351.2 | 271.3 | -60 | -24 | -6 | ^2^H_4_-PGD_2_ | 4.84 | 1.00 | 200 |
| 15-keto-PGF_1α_ | 353.3 | 193.1 | -40 | -38 | -6 | ^2^H_4_-PGE_2_ | 4.87 | 0.25 | 500 |
| 11,12-,15-TriHETrE | 353.2 | 167.1 | -80 | -28 | -10 | ^2^H_4_-PGE_2_ | 5.23 | 1.00 | 100 |
| LXA_4_ | 351.2 | 115.2 | -60 | -21 | -8 | ^2^H_4_-PGE_2_ | 5.25 | 0.18 | 70 |
| RvD_1_ | 375.3 | 141 | -50 | -20 | -8 | ^2^H_4_-PGE_2_ | 5.25 | 0.10 | 100 |
| 13,14-dihydro-15-keto-PGF_2α_ | 353.3 | 183.3 | -80 | -36 | -10 | ^2^H_4_-PGE_2_ | 5.44 | 0.10 | 500 |
| dihomo-PGF_2α_ | 381.4 | 221.1 | -60 | -38 | -10 | ^2^H_4_-PGE_2_ | 5.74 | 0.25 | 500 |
| 13,14-dihydro-15-keto-PGE_1_ | 353.3 | 221.2 | -40 | -30 | -6 | ^2^H_4_-PGE_2_ | 5.79 | 0.10 | 500 |
| RvE_2_ | 333.2 | 253.3 | -60 | -20 | -9 | ^2^H_4_-PGE_2_ | 6.04 | 1.00 | 100 |
| PGJ_2_ | 333.3 | 189.2 | -60 | -25 | -8 | ^2^H_4_-PGE_2_ | 6.57 | 1.60 | 160 |
| LTB_5_ | 333.3 | 195.2 | -65 | -22 | -8 | ^2^H_4_-LTB_4_ | 6.60 | 0.25 | 200 |
| PGB_2_ | 333.3 | 175.1 | -60 | -28 | -8 | ^2^H_4_-PGE_2_ | 6.69 | 0.40 | 800 |
| THF diol | 353.2 | 127.1 | -80 | -32 | -8 | ^2^H_4_-LTB_4_ | 6.78 | 0.25 | 100 |
| 18(S)-RvE_3_ | 333.2 | 201.3 | -60 | -20 | -9 | ^2^H_4_-PGE_2_ | 7.11 | 0.50 | 100 |
| 12-OH-17(18)-EpETE | 333.1 | 179.3 | -65 | -20 | -6 | ^2^H_4_-9,10-DiHOME | 7.19 | 0.25 | 100 |
| bicyclo PGE_2_ | 333.3 | 113.2 | -90 | -36 | -4 | ^2^H_4_-PGE_2_ | 7.28 | 1.00 | 500 |
| 15,16-DiHODE | 311.2 | 223.2 | -80 | -29 | -10 | ^2^H_4_-9,10-DiHOME | 7.37 | 0.50 | 400 |
| 9,10-DiHODE | 311.2 | 201.2 | -65 | -27 | -10 | ^2^H_4_-9,10-DiHOME | 7.41 | 0.20 | 400 |
| 8,15-DiHETE | 335.2 | 235.2 | -65 | -22 | -4 | ^2^H_11_-14,15-DiHETrE | 7.43 | 0.80 | 80 |
| 12,13-DiHODE | 311.2 | 183.1 | -80 | -30 | -8 | ^2^H_4_-9,10-DiHOME | 7.48 | 2.00 | 400 |
| 18(R)-RvE_3_ | 333.2 | 201.3 | -60 | -20 | -9 | ^2^H_4_-PGE_2_ | 7.66 | 1.00 | 100 |
| 6-trans-LTB4 | 335.2 | 195.1 | -65 | -23 | -9 | ^2^H_4_-LTB_4_ | 7.78 | 0.50 | 200 |
| 5,15-DiHETE | 335.3 | 173.2 | -60 | -21 | -8 | ^2^H_11_-14,15-DiHETrE | 7.79 | 0.25 | 100 |
| 17,18-DiHETE | 335.3 | 247.2 | -65 | -24 | -8 | ^2^H_11_-14,15-DiHETrE | 7.92 | 0.25 | 100 |
| LTB4 | 335.2 | 195.1 | -65 | -23 | -9 | ^2^H_4_-LTB4 | 8.20 | 0.50 | 200 |
| 14,15-DiHETE | 335.3 | 207.2 | -65 | -25 | -10 | ^2^H_11_-14,15-DiHETrE | 8.44 | 0.25 | 100 |
| 11,12-DiHETE | 335.2 | 167.1 | -65 | -26 | -5 | ^2^H_11_-14,15-DiHETrE | 8.64 | 0.25 | 100 |
| 12,13-DiHOME | 313.2 | 183.2 | -80 | -30 | -8 | ^2^H_4_-9,10-DiHOME | 8.83 | 1.25 | 1000 |
| 8,9-DiHETE | 335.2 | 127.1 | -65 | -26 | -5 | ^2^H_4_-9,10-DiHOME | 9.00 | 0.50 | 100 |
| 9,10-DiHOME | 313.2 | 201.2 | -80 | -29 | -8 | ^2^H_4_-9,10-DiHOME | 9.26 | 0.50 | 1000 |
| 14,15-DiHETrE | 337.2 | 207.1 | -65 | -25 | -10 | ^2^H_11_-14,15-DiHETrE | 9.88 | 0.25 | 200 |
| 19,20-DiHDPE | 361.2 | 273.2 | -65 | -24 | -6 | ^2^H_11_-14,15-DiHETrE | 9.90 | 1.00 | 100 |
| LTB_3_ | 337.2 | 195.2 | -65 | -22 | -8 | ^2^H_4_-LTB4 | 10.14 | 0.50 | 200 |
| 9,10-Dihydroxystearic acid | 315.0 | 170.8 | -60 | -36 | -9 | ^2^H_4_-9,10-DiHOME | 10.41 | 2.00 | 500 |
| 16,17-DiHDPE | 361.2 | 233.2 | -65 | -24 | -6 | ^2^H_11_-14,15-DiHETrE | 10.48 | 0.50 | 100 |
| 11,12-DiHETrE | 337.2 | 167.1 | -65 | -26 | -8 | ^2^H_11_-14,15-DiHETrE | 10.64 | 0.25 | 200 |
| 13,14-DiHDPE | 361.2 | 193.2 | -65 | -24 | -6 | ^2^H_11_-14,15-DiHETrE | 10.73 | 0.25 | 100 |
| 20-HEPE | 317.2 | 287.3 | -50 | -20 | -8 | ^2^H_8_-12-HETE | 10.83 | 0.50 | 100 |
| 9-HOTrE | 293.2 | 171.2 | -65 | -22 | -8 | _2_H^4^-9-HODE | 11.01 | 0.50 | 100 |
| 10,11-DiHDPE | 361.2 | 153.2 | -65 | -24 | -6 | ^2^H_11_-14,15-DiHETrE | 11.11 | 0.50 | 100 |
| 8,9-DiHETrE | 337.2 | 127.1 | -70 | -30 | -8 | ^2^H_11_-14,15-DiHETrE | 11.26 | 0.50 | 200 |
| EKODE | 309.2 | 291.1 | -65 | -20 | -6 | ^2^H_4_-9-HODE | 11.33 | 0.50 | 100 |
| 13-HOTrE | 293.2 | 195.1 | -70 | -24 | -8 | ^2^H_4_-9-HODE | 11.34 | 0.60 | 60 |
| 18-HEPE | 317.2 | 259.2 | -55 | -17 | -7 | ^2^H_4_-9-HODE | 11.40 | 0.10 | 100 |
| 5,6-DiHETE | 335.2 | 115.2 | -60 | -21 | -8 | ^2^H_11_-14,15-DiHETrE | 11.79 | 0.25 | 100 |
| 15-deoxy-PGJ_2_ | 315.2 | 271.2 | -65 | -20 | -6 | ^2^H_11_-14,15-DiHETrE | 11.84 | 1.00 | 400 |
| 7,8-DiHDPE | 361.2 | 113.1 | -65 | -24 | -6 | ^2^H_11_-14,15-DiHETrE | 11.91 | 1.00 | 100 |
| 15-HEPE | 317.2 | 219.2 | -60 | -20 | -10 | ^2^H_8_-12-HETE | 12.08 | 1.25 | 500 |
| 20-HETE | 319.2 | 275.1 | -80 | -23 | -6 | ^2^H_6_-20-HETE | 12.10 | 2.60 | 260 |
| 5,6-DiHETrE | 337.2 | 145.1 | -70 | -26 | -10 | ^2^H_11_-14,15-DiHETrE | 12.17 | 0.50 | 200 |
| 8-HEPE | 317.2 | 155.2 | -60 | -20 | -8 | ^2^H_8_-12-HETE | 12.42 | 0.63 | 500 |
| 12-HEPE | 317.2 | 179.2 | -65 | -20 | -8 | ^2^H_8_-12-HETE | 12.59 | 0.63 | 500 |
| 5-HEPE | 317.2 | 115.1 | -60 | -20 | -6 | ^2^H_8_-12-HETE | 13.12 | 0.50 | 400 |
| 4,5-DiHDPE | 361.2 | 229.3 | -65 | -24 | -6 | ^2^H_11_-14,15-DiHETrE | 13.13 | 2.00 | 100 |
| 13-HODE | 295.2 | 195.2 | -80 | -26 | -9 | ^2^H_4_-9-HODE | 13.33 | 1.00 | 2000 |
| 9-HODE | 295.2 | 171.1 | -80 | -26 | -7 | ^2^H_4_-9-HODE | 13.44 | 1.00 | 2000 |
| 20-HDHA | 343.2 | 241.1 | -55 | -19 | -7 | ^2^H_8_-12-HETE | 13.72 | 0.25 | 20 |
| 15(16)-EpODE | 293.3 | 235.2 | -65 | -20 | -4 | ^2^H_4_-9(10)-EpOME | 13.96 | 0.25 | 100 |
| 15-HETE | 319.2 | 219.2 | -60 | -20 | -8 | ^2^H_8_-12-HETE | 14.02 | 1.25 | 1000 |
| 9(10)-EpODE | 293.3 | 171.2 | -65 | -20 | -8 | ^2^H_4_-9(10)-EpOME | 14.11 | 0.20 | 80 |
| 17(18)-EpETE | 317.2 | 215.2 | -65 | -20 | -6 | ^2^H_11_-14(15)-EpETrE | 14.15 | 0.50 | 100 |
| 16-HDHA | 343.2 | 233.2 | -55 | -19 | -7 | ^2^H_8_-12-HETE | 14.21 | 0.10 | 20 |
| 17-HDHA | 343.2 | 201.2 | -60 | -20 | -6 | ^2^H_8_-12-HETE | 14.33 | 0.50 | 100 |
| 13-HDHA | 343.2 | 193.1 | -55 | -19 | -7 | ^2^H_8_-12-HETE | 14.47 | 0.10 | 20 |
| 12(13)-EpODE | 293.2 | 183.1 | -65 | -24 | -8 | ^2^H_4_-9(10)-EpOME | 14.50 | 0.25 | 100 |
| 13-oxo-ODE | 293.2 | 195.1 | -75 | -20 | -8 | ^2^H_4_-9-HODE | 14.50 | 1.00 | 100 |
| 11-HETE | 319.2 | 167.2 | -60 | -23 | -7 | ^2^H_8_-12-HETE | 14.52 | 0.50 | 1000 |
| 10-HDHA | 343.2 | 153.2 | -45 | -21 | -7 | ^2^H_8_-12-HETE | 14.68 | 0.10 | 20 |
| 14-HDHA | 343.2 | 205.2 | -50 | -19 | -7 | ^2^H_8_-12-HETE | 14.68 | 0.25 | 500 |
| 15-oxo-ETE | 317.2 | 113.1 | -65 | -25 | -8 | ^2^H_8_-5-HETE | 14.70 | 0.50 | 100 |
| 9-oxo-ODE | 293.2 | 185.1 | -90 | -28 | -8 | ^2^H_4_-9-HODE | 14.73 | 1.00 | 100 |
| 14(15)-EpETE | 317.2 | 207.2 | -65 | -20 | -6 | ^2^H_11_-14(15)-EpETrE | 14.78 | 0.25 | 100 |
| 8-HETE | 319.2 | 155.2 | -60 | -22 | -6 | ^2^H_8_-12-HETE | 14.86 | 2.50 | 1000 |
| 12-HETE | 319.2 | 179.2 | -60 | -20 | -8 | ^2^H_8_-12-HETE | 14.88 | 0.50 | 1000 |
| 11(12)-EpETE | 317.2 | 167.2 | -65 | -20 | -6 | ^2^H_11_-14(15)-EpETrE | 14.91 | 0.50 | 100 |
| 11-HDHA | 343.2 | 121.1 | -45 | -20 | -7 | ^2^H_8_-5-HETE | 14.93 | 0.10 | 20 |
| 7-HDHA | 343.2 | 141.2 | -55 | -19 | -7 | ^2^H_8_-5-HETE | 15.06 | 0.25 | 500 |
| 8(9)-EpETE | 317.2 | 127.2 | -65 | -20 | -6 | ^2^H_11_-14(15)-EpETrE | 15.07 | 1.00 | 100 |
| 9-HETE | 319.2 | 167.2 | -60 | -23 | -7 | ^2^H_8_-5-HETE | 15.16 | 2.50 | 1000 |
| 15(S)-HETrE | 321.2 | 221.2 | -70 | -23 | -10 | ^2^H_8_-5-HETE | 15.19 | 0.50 | 200 |
| 8-HDHA | 343.2 | 189.2 | -50 | -19 | -7 | ^2^H_8_-5-HETE | 15.22 | 0.10 | 20 |
| 5-HETE | 319.2 | 115.2 | -60 | -21 | -7 | ^2^H_8_-5-HETE | 15.38 | 1.25 | 1000 |
| 4-HDHA | 343.2 | 101.1 | -55 | -19 | -7 | ^2^H_8_-5-HETE | 15.78 | 0.10 | 500 |
| 19(20)-EpDPE | 343.2 | 241.2 | -65 | -20 | -7 | ^2^H_11_-14(15)-EpETrE | 15.83 | 0.25 | 100 |
| 12(13)-EpOME | 295.3 | 195.2 | -80 | -23 | -8 | ^2^H_4_-9(10)-EpOME | 15.91 | 0.25 | 200 |
| 14(15)-EpETrE | 319.2 | 219.3 | -65 | -20 | -4 | 2H11-14(15)-EpETrE | 16.03 | 0.50 | 100 |
| 9(10)-EpOME | 295.3 | 171.1 | -80 | -23 | -8 | ^2^H_4_-9(10)-EpOME | 16.08 | 0.25 | 200 |
| 16(17)-EpDPE | 343.2 | 233.2 | -65 | -20 | -7 | ^2^H_11_-14(15)-EpETrE | 16.20 | 0.25 | 100 |
| 13(14)-EpDPE | 343.2 | 193.2 | -65 | -20 | -7 | ^2^H_11_-14(15)-EpETrE | 16.28 | 0.50 | 100 |
| 5-oxo-ETE | 317.2 | 273.2 | -65 | -22 | -6 | ^2^H_4_-9(10)-EpOME | 16.36 | 2.00 | 100 |
| 10(11)-EpDPE | 343.2 | 153.2 | -65 | -20 | -7 | ^2^H_11_-14(15)-EpETrE | 16.36 | 0.25 | 100 |
| 11(12)-EpETrE | 319.3 | 167.2 | -60 | -20 | -7 | ^2^H_11_-14(15)-EpETrE | 16.48 | 0.50 | 200 |
| 8(9)-EpETrE | 319.2 | 155.2 | -65 | -20 | -6 | ^2^H_11_-14(15)-EpETrE | 16.63 | 2.00 | 100 |
| 5(6)-EpETrE | 319.2 | 191.1 | -60 | -20 | -7 | ^2^H_11_-14(15)-EpETrE | 16.78 | 1.00 | 100 |
| 9(10)-Epoxystearic acid | 297.0 | 170.8 | -100 | -28 | -11 | ^2^H_4_-9(10)-EpOME | 17.25 | 1.00 | 100 |

^*^ LLOQ was set to the lowest calibration standard injected yielding a signal to noise ratio ≥ 9 and an accuracy within ± 20%.

^†^ ULOQ does not reflect the end of the linear range but the concentration of the highest calibrator.
