# Supplementary material for: Food Polyphenols Fail to Cause a Biologically Relevant Reduction of COX-2 Activity
Source: PLoS One. 2015 Oct 6;10(10):e0139147. doi: 10.1371/journal.pone.0139147 (PMC4594923; doi:10.1371/journal.pone.0139147)
Supplement: S2 Table — (DOCX) [file pone.0139147.s003.docx]

**Table S2:** Oxylipin concentrations in plasma 24 h after LPS treatment.

| **COXi** | **-** | | **-** | | **Celecoxib** | | **Apigenin** | | **Genistein** | | **Resveratrol** | | **ε-viniferin** | |
| --- | --- | --- | --- | --- | --- | --- | --- | --- | --- | --- | --- | --- | --- | --- |
| **LPS** | **-** | | **+** | | **+** | | **+** | | **+** | | **+** | | **+** | |
| **Analyte (nM)** | **Mean** | **SE** | **Mean** | **SE** | **Mean** | **SE** | **Mean** | **SE** | **Mean** | **SE** | **Mean** | **SE** | **Mean** | **SE** |
| TXB_2_ | 2.4 | 0.67 | 1.9 | 0.73 | 0.80 | 0.191 | 1.1 | 0.25 | 2.6 | 0.77 | 1.1 | 0.075 | 4.2 | 0.75 |
| PGE_2_ | 0.33^*^ | 0.19 | 1.6 | 0.23 | 0.079^†^ | 0.016 | 1.3 | 0.36 | 0.95 | 0.28 | 3.5^†^ | 0.52 | 1.6 | 0.50 |
| 6-keto-PGF_1α_ | < 0.45 | - | 1.8 | 0.18 | < 0.45 | - | 2.1 | 0.22 | 2.4 | 0.31 | 2.5 | 0.30 | 2.8 | 0.67 |
| PGF_2α_ | 0.40 | 0.023 | 0.44 | 0.030 | 0.35 | - | 0.42 | 0.059 | 0.44 | 0.051 | 0.51 | 0.050 | 0.77^‡^ | 0.10 |
| PGD_2_ | < 0.50 | - | < 0.50 | - | < 0.50 | - | < 0.50 | - | < 0.50 | - | < 0.50 | - | 0.90 | 0.13 |
| 5-iPF_2α_ | < 0.50 | - | < 0.50 | - | 0.63 | 0.078 | < 0.50 | - | < 0.50 | - | < 0.50 | - | 0.91^‡^ | 0.12 |
| 1a,1b-dihomo-PGF­_2α_ | 0.20 | 0.020 | 0.22 | 0.013 | 0.28 | 0.043 | 0.27 | 0.018 | 0.33 | 0.054 | 0.21 | 0.021 | 0.42^†^ | 0.061 |
| 13,14dihydro-15-keto-PGF_2α_ | 0.30^*^ | 0.062 | 0.64 | 0.015 | 0.13^†^ | 0.033 | 0.41 | 0.12 | 0.43 | 0.091 | 0.70 | 0.046 | 2.1^‡^ | 0.23 |
| PGF_1α_ | 0.11 | 0.015 | 0.10 | 0.017 | 0.073 | 0.013 | 0.10 | 0.025 | 0.13 | 0.032 | 0.088 | 0.011 | 0.25^‡^ | 0.029 |
| 13,14-dihydro-15-keto-PGE_1_ | < 0.05 | - | 0.17 | 0.025 | < 0.050 | - | 0.14 | 0.022 | 0.18 | 0.037 | 0.22 | 0.0087 | 0.34^†^ | 0.066 |
| 8-iPF_2a_ | 0.10 | 0.015 | 0.17 | 0.0079 | 0.19 | 0.052 | 0.19 | 0.021 | 0.24 | 0.049 | 0.16 | 0.0053 | 0.30^*^ | 0.037 |
| 9-HOTrE | 33 | 8.1 | 15 | 7.8 | 7.5 | 1.5 | 6.1 | 1.6 | 4.1 | 0.50 | 3.6 | 0.28 | 12 | 8.1 |
| 13-HOTrE | 44 | 11 | 25 | 11 | 18 | 4.5 | 5.7 | 0.82 | 15 | 5.2 | 11 | 2.4 | 61 | 32 |
| 20-HETE | 1.3 | 0.013 | 1.4 | 0.039 | 1.7 | 0.22 | 1.6 | 0.18 | 1.7 | 0.12 | 1.5 | 0.11 | 2.7^‡^ | 0.19 |
| 15-HEPE | < 0.63 | - | 0.68 | 0.028 | 0.88 | 0.14 | 0.63 | - | 0.96 | 0.21 | 0.63 | - | 1.5^‡^ | 0.30 |
| 8-HEPE | < 0.31 | - | 0.46 | 0.042 | 0.65 | 0.17 | 0.31 | - | 0.72 | 0.21 | 0.56 | 0.16 | 1.2^†^ | 0.22 |
| 12-HEPE | 20 | 7.6 | 59 | 10 | 131 | 49 | 25 | 12 | 140 | 61 | 75 | 24 | 239^†^ | 50 |
| 5-HEPE | 0.34 | 0.017 | 0.37 | 0.017 | 0.25 | - | 0.27 | 0.0091 | 0.33 | 0.021 | 0.29 | 0.014 | 0.25^*^ | - |
| 13-HODE | 490 | 83 | 300 | 95 | 264 | 51 | 110^*^ | 15 | 240 | 66 | 150.00 | 24 | 540 | 181 |
| 9-HODE | 290 | 52 | 170 | 61 | 109 | 15 | 83 | 13 | 96 | 16 | 73 | 3.0 | 210 | 85 |
| 15-HETE | 2.6 | 0.45 | 4.0 | 0.32 | 3.9 | 0.90 | 2.5 | 0.27 | 5.1 | 1.3 | 3.4 | 0.39 | 7.9^†^ | 1.2 |
| 11-HETE | 1.5^*^ | 0.24 | 2.6 | 0.16 | 1.5 | 0.30 | 1.8 | 0.12 | 2.7 | 0.39 | 2.8 | 0.20 | 3.8^*^ | 0.50 |
| 8-HETE | 2.7 | 0.49 | 4.9 | 0.52 | 8.5 | 2.3 | 3.1 | 0.76 | 9.6 | 3.4 | 5.1 | 1.2 | 12^*^ | 1.5 |
| 12-HETE | 230 | 60 | 480 | 60 | 1100 | 350 | 260 | 100 | 1200 | 490 | 520 | 132 | 1700^†^ | 210 |
| 15(S)-HETrE | 0.84 | 0.16 | 1.6 | 0.18 | 2.2 | 0.61 | 0.94 | 0.17 | 2.7 | 0.85 | 1.7 | 0.31 | 5.7^‡^ | 1.14 |
| 5-HETE | 2.4 | 0.24 | 3.1 | 0.15 | 2.4 | 0.30 | 2.4 | 0.30 | 3.1 | 0.17 | 2.8 | 0.10 | 2.8 | 0.16 |
| 20-HEPE | 2.0^*^ | 0.22 | 3.6 | 0.24 | 1.8^†^ | 0.44 | 1.7^*^ | 0.22 | 2.8 | 0.27 | 2.7 | 0.48 | 5.7^†^ | 0.75 |
| 18-HEPE | 0.41 | 0.043 | 0.52 | 0.035 | 0.32 | 0.061 | 0.26 | 0.024 | 0.45 | 0.026 | 0.44 | 0.049 | 0.81 | 0.35 |
| 20-HDHA | 1.9 | 0.36 | 3.1 | 0.27 | 3.0 | 0.67 | 2.2 | 0.19 | 3.7 | 0.77 | 2.2 | 0.12 | 3.6 | 0.15 |
| 16-HDHA | 0.78^*^ | 0.18 | 1.6 | 0.14 | 1.1 | 0.25 | 1.0 | 0.062 | 1.9 | 0.33 | 1.1 | 0.10 | 1.1 | 0.068 |
| 13-HDHA | 0.72 | 0.18 | 1.7 | 0.17 | 2.3 | 0.68 | 0.98 | 0.13 | 2.8 | 1.0 | 1.7 | 0.32 | 3.9^*^ | 0.68 |
| 17-HDHA | 4.2 | 1.24 | 10 | 1.4 | 21 | 7.2 | 5.2 | 1.2 | 21 | 11 | 12 | 3.8 | 39^†^ | 7.1 |
| 10-HDHA | 2.3 | 0.74 | 6.8 | 1.1 | 16 | 5.5 | 3.0 | 1.1 | 16 | 7.8 | 9.7 | 3.2 | 27^†^ | 5.4 |
| 14-HDHA | 56 | 20 | 170 | 28 | 470 | 170.0 | 71 | 30 | 450 | 240 | 270 | 94 | 800^†^ | 170 |
| 11-HDHA | 0.53 | 0.10 | 0.92 | 0.084 | 1.0 | 0.23 | 0.67 | 0.046 | 1.2 | 0.36 | 0.61 | 0.051 | 0.91 | 0.089 |
| 7-HDHA | 0.41 | 0.064 | 0.62 | 0.044 | 0.52 | 0.11 | 0.47 | 0.045 | 0.64 | 0.11 | 0.35 | 0.024 | 0.55 | 0.042 |
| 8-HDHA | 2.4 | 0.28 | 3.6 | 0.21 | 3.8 | 0.62 | 3.0 | 0.17 | 4.0 | 0.63 | 2.9 | 0.13 | 3.8 | 0.19 |
| 4-HDHA | 1.1 | 0.20 | 1.7 | 0.16 | 1.9 | 0.45 | 1.3 | 0.096 | 2.0 | 0.35 | 1.1 | 0.029 | 1.7 | 0.070 |
| 9,12,13-TriHOME | 18 | 2.0 | 14 | 2.2 | 22 | 4.2 | 38 | 20 | 9.6 | 2.6 | 11 | 2.7 | 17 | 7.1 |
| 9,10,13-TriHOME | 3.2 | 0.28 | 2.5 | 0.29 | 2.7 | 0.24 | 3.3 | 1.2 | 1.5 | 0.30 | 1.6 | 0.24 | 2.2 | 0.54 |
| 13-oxo-ODE | 11 | 1.9 | 7.1 | 2.2 | 2.7 | 0.62 | 2.5 | 0.34 | 2.7 | 0.24 | 2.7 | 0.12 | 8.3 | 3.1 |
| 15-oxo-ETE | 0.35 | 0.05 | 0.39 | 0.024 | 0.39 | 0.089 | 0.29 | 0.033 | 0.42 | 0.068 | 0.28 | 0.013 | 0.34 | 0.023 |
| 9-oxo-ODE | 42 | 5.8 | 31 | 9.0 | 18 | 2.9 | 16 | 2.7 | 16 | 1.7 | 16 | 0.89 | 38 | 16 |
| 5-oxo-ETE | 3.4 | 0.65 | 2.8 | 0.28 | < 1.0 | - | < 1.0 | - | 1.2 | 0.13 | 1.0 | - | 4.7 | 0.71 |
| EKODE | 9.3 | 1.4 | 11 | 1.0 | 12 | 2.1 | 7.0 | 0.73 | 9.9 | 1.1 | 11 | 1.6 | 16 | 3.4 |
| 9(10)-EpODE | 4.3 | 0.73 | 5.5 | 0.41 | 2.6^*^ | 0.99 | 2.2^*^ | 0.31 | 3.5 | 0.50 | 2.2^*^ | 0.17 | 6.5 | 0.18 |
| 17(18)-EpETE | 0.70^†^ | 0.14 | 1.4 | 0.085 | 0.66^†^ | 0.076 | 0.68^*^ | 0.12 | 1.2 | 0.28 | 1.1 | 0.073 | 1.1 | 0.056 |
| 12(13)-EpODE | 6.7 | 0.64 | 4.6 | 1.4 | 1.8 | 0.51 | 1.8 | 0.38 | 1.8 | 0.18 | 1.4 | 0.042 | 3.3 | 0.024 |
| 14(15)-EpETE | 0.25 | 0.045 | 0.30 | 0.019 | 0.13 | - | 0.14^†^ | 0.0065 | 0.21 | 0.021 | 0.16^*^ | 0.0085 | 0.18^*^ | 0.015 |
| 19(20)-EpDPE | 6.8^†^ | 1.5 | 14 | 1.0 | 10 | 0.96 | 9.4 | 1.1 | 14 | 1.8 | 13 | 1.1 | 13 | 0.90 |
| 12(13)-EpOME | 70 | 4.1 | 60 | 10.13 | 24^†^ | 6.8 | 29^*^ | 5.3 | 34 | 3.3 | 23^†^ | 1.1 | 48 | 1.7 |
| 14(15)-EpETrE | 2.6 | 0.36 | 2.8 | 0.11 | 1.2^†^ | 0.40 | 1.5^*^ | 0.12 | 2.0 | 0.19 | 1.5^*^ | 0.089 | 2.4 | 0.21 |
| 9(10)-EpOME | 36 | 7.3 | 50 | 2.3 | 24^*^ | 8.1 | 24^*^ | 3.2 | 37 | 5.0 | 24^*^ | 1.1 | 61 | 1.7 |
| 16(17)-EpDPE | 1.8 | 0.40 | 2.6 | 0.096 | 1.3 | 0.53 | 1.2 | 0.042 | 2.1 | 0.36 | 1.3 | 0.061 | 2.8 | 0.21 |
| 13(14)-EpDPE | 1.7 | 0.39 | 2.4 | 0.099 | 1.3 | 0.53 | 1.2 | 0.036 | 2.0 | 0.34 | 1.3 | 0.071 | 2.9 | 0.23 |
| 10(11)-EpDPE | 2.4 | 0.56 | 3.5 | 0.14 | 2.0 | 0.74 | 1.7 | 0.077 | 3.0 | 0.58 | 1.9 | 0.11 | 3.9 | 0.30 |
| 11(12)-EpETrE | 3.6 | 0.51 | 3.4 | 0.14 | 1.7^*^ | 0.57 | 2.0 | 0.13 | 2.7 | 0.37 | 1.7 | 0.028 | 2.6 | 0.25 |
| 8(9)-EpETrE | 1.9 | 0.31 | 2.4 | 0.15 | 2.0 | 0.55 | 1.5 | 0.21 | 2.3 | 0.31 | 1.7 | 0.19 | 2.5 | 0.27 |
| 5(6)-EpETrE | 8.3 | 1.3 | 10 | 0.24 | 6.9 | 1.3 | 6.8 | 1.2 | 8.9 | 0.91 | 7.0 | 0.088 | 11 | 0.85 |
| 15(16)-EpODE | 1.7 | 0.39 | 2.4 | 0.099 | 1.3 | 0.53 | 1.2 | 0.036 | 2.0 | 0.34 | 1.3 | 0.071 | 2.9 | 0.23 |
| 9(10)-Epoxystearic acid | 26 | 3.7 | 29 | 1.2 | 23 | 6.1 | 18 | 1.8 | 31 | 4.9 | 17 | 0.93 | 37 | 1.1 |
| 15,16-DiHODE | 44 | 3.8 | 39 | 4.1 | 43 | 5.8 | 24 | 4.9 | 40 | 8.4 | 26 | 1.8 | 53 | 5.8 |
| 9,10-DiHODE | 7.2 | 0.94 | 6.5 | 1.1 | 3.9 | 0.38 | 4.0 | 0.84 | 4.8 | 1.1 | 3.4 | 0.43 | 4.5 | 0.34 |
| 12,13-DiHODE | 9.7 | 1.3 | 7.5 | 1.4 | 4.1 | 0.53 | 4.5 | 1.1 | 4.7 | 1.5 | 3.6 | 0.78 | 2.8 | 0.20 |
| 17,18-DiHETE | 1.2^†^ | 0.12 | 2.3 | 0.16 | 0.79^‡^ | 0.18 | 1.2^*^ | 0.11 | 2.3 | 0.39 | 1.8 | 0.31 | 1.8 | 0.37 |
| 14,15-DiHETE | 0.26^*^ | 0.029 | 0.46 | 0.031 | < 0.13 | - | 0.19^†^ | 0.020 | 0.41 | 0.11 | 0.32 | 0.079 | 0.36 | 0.026 |
| 11,12-DiHETE | < 0.13 | - | 0.22 | 0.016 | < 0.13 | - | < 0.13 | - | 0.19 | 0.030 | 0.17 | 0.022 | 0.38^‡^ | 0.062 |
| 12,13-DiHOME | 180 | 18 | 230 | 16 | 110^*^ | 8.0 | 114 | 17 | 214 | 69 | 130 | 22 | 110^*^ | 7.3 |
| 9,10-DiHOME | 46 | 5.0 | 56 | 3.8 | 33 | 2.9 | 34 | 4.3 | 63 | 17 | 36 | 3.4 | 30 | 1.7 |
| 19,20-DiHDPE | 7.6^†^ | 1.4 | 17 | 1.4 | 6.1^‡^ | 1.2 | 11 | 0.87 | 19 | 2.8 | 14 | 2.0 | 14 | 1.8 |
| 14,15-DiHETrE | 2.2^*^ | 0.33 | 4.4 | 0.39 | 1.1^‡^ | 0.20 | 2.2 | 0.32 | 4.3 | 1.3 | 2.8 | 0.66 | 2.5 | 0.24 |
| 16,17-DiHDPE | 1.1^†^ | 0.17 | 2.4 | 0.20 | 0.76^‡^ | 0.12 | 1.2^*^ | 0.13 | 2.2 | 0.52 | 1.7 | 0.37 | 1.1^†^ | 0.078 |
| 11,12-DiHETrE | 1.0^†^ | 0.15 | 2.1 | 0.18 | 0.83^†^ | 0.21 | 1.1^*^ | 0.12 | 1.9 | 0.41 | 1.4 | 0.29 | 2.0 | 0.17 |
| 13,14-DiHDPE | 0.50^†^ | 0.073 | 1.0 | 0.076 | 0.39^‡^ | 0.072 | 0.58^*^ | 0.062 | 0.96 | 0.18 | 0.73 | 0.14 | 0.55^*^ | 0.048 |
| 10,11-DiHDPE | 0.49^‡^ | 0.065 | 1.0 | 0.080 | 0.40^‡^ | 0.064 | 0.58^*^ | 0.0671 | 0.90 | 0.16 | 0.66 | 0.11 | 0.62^*^ | 0.054 |
| 8,9-DiHETrE | 1.5^*^ | 0.17 | 2.3 | 0.15 | 1.3^†^ | 0.22 | 1.4 | 0.16 | 2.1 | 0.29 | 1.6 | 0.20 | 2.5 | 0.15 |
| 7,8-DiHDPE | 0.74 | 0.055 | 1.1 | 0.076 | 0.67^*^ | 0.12 | 0.64 | 0.042 | 1.1 | 0.16 | 0.71 | 0.091 | 1.1 | 0.11 |
| 5,6-DiHETrE | 0.59 | 0.047 | 0.71 | 0.039 | 0.61 | 0.082 | 0.62 | 0.10 | 0.72 | 0.063 | 0.65 | 0.021 | 1.3^‡^ | 0.12 |
| 4,5-DiHDPE | 3.9 | 0.59 | 7.5 | 0.60 | 12 | 1.4 | 7.8 | 1.1 | 10 | 1.3 | 8.6 | 0.58 | 23^‡^ | 3.9 |
| 9,10-Dihydroxystearic acid | 18 | 1.8 | 11 | 1.3 | 32^†^ | 5.4 | 47^‡^ | 7.4 | 15 | 2.3 | 20 | 4.4 | 10 | 1.2 |

If analyte concentration is below the limit of quantification (LOQ), it is indicated as <. The given value represents the LOQ.

^*^ p <0.05 Dunnetts test vs. LPS

^†^ p <0.01 Dunnetts test vs. LPS

^‡^ p <0.001 Dunnetts test vs. LPS
