# Supplementary material for: Food Polyphenols Fail to Cause a Biologically Relevant Reduction of COX-2 Activity
Source: PLoS One. 2015 Oct 6;10(10):e0139147. doi: 10.1371/journal.pone.0139147 (PMC4594923; doi:10.1371/journal.pone.0139147)
Supplement: S3 Table — (DOCX) [file pone.0139147.s004.docx]

**Table S3:** Oxylipin concentrations in kidney tissue 24 h after LPS treatment.

| **COXi** | **-** | | **-** | | **Celecoxib** | | **Apigenin** | | **Genistein** | | **Resveratrol** | | **ε-viniferin** | |
| --- | --- | --- | --- | --- | --- | --- | --- | --- | --- | --- | --- | --- | --- | --- |
| **LPS** | **-** | | **+** | | **+** | | **+** | | **+** | | **+** | | **+** | |
| **Analyte (nM)** | **Mean** | **SE** | **Mean** | **SE** | **Mean** | **SE** | **Mean** | **SE** | **Mean** | **SE** | **Mean** | **SE** | **Mean** | **SE** |
| TXB_2_ | 1.5 | 0.24 | 1.5 | 0.19 | 1.3 | 0.22 | 2.0 | 0.12 | 3.2^*^ | 0.21 | 3.8^†^ | 0.56 | 4.9^‡^ | 0.66 |
| PGF_2α_ | 21 | 2.7 | 30 | 3.3 | 15 | 2.8 | 33 | 2.4 | 40 | 4.1 | 70^‡^ | 11 | 63^‡^ | 5.2 |
| PGE_2_ | 55 | 8.5 | 81 | 13 | 45 | 8.7 | 110 | 19 | 110 | 23 | 210^‡^ | 36 | 110 | 20 |
| 6-keto-PGF_1α_ | 31 | 3.4 | 41 | 4.3 | 36 | 8.7 | 55 | 3.4 | 61 | 7.2 | 80^*^ | 14 | 108^‡^ | 12 |
| PGF_2α_ | 21 | 2.7 | 30 | 3.3 | 15 | 2.8 | 33 | 2.4 | 40 | 4.1 | 70 | 11 | 63 | 5.2 |
| PGE_1_ | 2.0 | 0.33 | 5.0 | 0.73 | 2.5 | 0.42 | 4.7 | 0.49 | 6.3 | 1.4 | 11^†^ | 2.1 | 8.3 | 1.0 |
| PGD_1_ | < 0.50 | - | < 0.5 | - | < 0.50 | - | < 0.50 | - | < 0.50 | - | 0.72 | 0.093 | 0.84^†^ | 0.12 |
| PGD_2_ | 8.4 | 1.2 | 9.0 | 1.4 | 7.4 | 1.0 | 13 | 1.6 | 9.6 | 1.4 | 20^†^ | 3.0 | 19^†^ | 3.4 |
| 5-iPF_2α_ | < 1.0 | - | < 1.0 | - | < 1.0 | - | < 1.0 | - | < 1.0 | - | < 1.0 | - | 1.5^‡^ | 0.18 |
| 1a,1b-dihomo-PGF_2α_ | 0.57^*^ | 0.11 | 1.7 | 0.29 | 0.54^*^ | 0.16 | 1.3 | 0.19 | 1.9 | 0.28 | 3.9^‡^ | 0.50 | 3.2^†^ | 0.39 |
| 13,14-dihydro-15-keto-PGF_2α_ | 1.8 | 0.17 | 2.3 | 0.19 | 0.72^†^ | 0.14 | 2.7 | 0.18 | 3.1 | 0.12 | 4.3^‡^ | 0.50 | 4.6^‡^ | 0.50 |
| PGF_1α_ | 1.8^*^ | 0.21 | 3.4 | 0.48 | 1.4^†^ | 0.23 | 2.8 | 0.40 | 3.7 | 0.38 | 6.2^†^ | 0.65 | 7.7^‡^ | 0.44 |
| 13,14-dihydro-15-keto-PGE_1_ | 0.22 | 0.038 | 0.44 | 0.049 | < 0.1 | - | 0.41 | 0.046 | 0.5 | 0.13 | 0.6 | 0.10 | 0.49 | 0.042 |
| 8-iPF_2a_ | 1.2 | 0.089 | 1.5 | 0.16 | 1.2 | 0.17 | 1.8 | 0.16 | 2.0 | 0.09 | 3.2^‡^ | 0.37 | 2.7^†^ | 0.31 |
| 9-HOTrE | 6.6 | 1.3 | 4.1 | 0.44 | 4.3 | 0.40 | 3.2 | 0.49 | 5.0 | 0.75 | 3.9 | 0.46 | 5.8 | 0.22 |
| 13-HOTrE | 8.4^*^ | 1.4 | 4.5 | 0.35 | 5.2 | 0.51 | 3.3 | 0.51 | 6.0 | 1.1 | 3.6 | 0.61 | 6.0 | 0.45 |
| 20-HETE | < 2.7 | - | < 2.7 | - | < 2.7 | - | < 2.7 | - | < 2.7 | - | < 2.7 | - | 3.6^*^ | 0.48 |
| 12-HEPE | 3.7 | 1.2 | 1.8 | 0.46 | 2.9 | 0.85 | 1.1 | 0.093 | 2.2 | 0.66 | 2.1 | 0.55 | 5.0 | 1.2 |
| 5-HEPE | 0.87 | 0.038 | 0.92 | 0.082 | 1.5^‡^ | 0.13 | 1.0 | 0.11 | 1.3 | 0.088 | 1.1 | 0.12 | 1.1 | 0.036 |
| 13-HODE | 170 | 27 | 170 | 23 | 140 | 14 | 120 | 18 | 230 | 35 | 270 | 43 | 310^†^ | 20 |
| 9-HODE | 160 | 23 | 200 | 28 | 160 | 17 | 150 | 24 | 280 | 46 | 350^*^ | 52 | 380^†^ | 33 |
| 15-HETE | 18 | 1.5 | 21 | 1.5 | 19 | 1.1 | 22 | 1.6 | 31^*^ | 2.5 | 38^‡^ | 2.9 | 30^*^ | 2.1 |
| 11-HETE | 9.8 | 1.1 | 13 | 1.2 | 10 | 1.2 | 16 | 1.8 | 18 | 1.6 | 31^‡^ | 3.1 | 22^†^ | 3.1 |
| 8-HETE | 3.8 | 0.21 | 3.6 | 0.22 | 4.6 | 0.27 | 3.4 | 0.33 | 4.9 | 0.63 | 4.2 | 0.46 | 4.4 | 0.10 |
| 12-HETE | 43 | 16 | 17 | 3.2 | 29 | 9.6 | 10 | 0.91 | 21 | 4.4 | 24 | 6.6 | 47 | 11 |
| 9-HETE | 3.0 | 0.16 | 3.1 | 0.20 | 4.0^*^ | 0.16 | 3.5 | 0.24 | 4.3^†^ | 0.37 | 3.1 | 0.20 | 3.6 | 0.19 |
| 15(S)-HETrE | 3.7 | 0.24 | 4.7 | 0.40 | 4.0 | 0.22 | 4.6 | 0.34 | 6.4 | 0.38 | 7.4^‡^ | 0.74 | 8.0^‡^ | 0.36 |
| 5-HETE | 10 | 0.72 | 9.9 | 0.90 | 16^‡^ | 0.60 | 12 | 1.2 | 15^†^ | 0.71 | 12 | 1.0 | 13 | 0.48 |
| 20-HEPE | 7.0 | 0.40 | 7.1 | 0.54 | 5.3 | 1.7 | 5.2 | 0.75 | 5.9 | 0.45 | 5.3 | 0.76 | 11 | 2.1 |
| 18-HEPE | 1.0 | 0.10 | 1.4 | 0.16 | 1.3 | 0.052 | 1.5 | 0.14 | 2.0^*^ | 0.19 | 1.3 | 0.092 | 1.5 | 0.11 |
| 20-HDHA | 13 | 1.3 | 18 | 1.7 | 18 | 0.82 | 18 | 1.7 | 25^*^ | 2.0 | 19 | 1.7 | 17 | 0.90 |
| 16-HDHA | 4.6^†^ | 0.29 | 7.0 | 0.57 | 6.1 | 0.45 | 6.3 | 0.46 | 9.1 | 0.78 | 6.9 | 0.65 | 4.9^*^ | 0.32 |
| 13-HDHA | 4.3^†^ | 0.30 | 6.7 | 0.43 | 6.0 | 0.52 | 6.9 | 0.45 | 9.1^*^ | 0.70 | 11^‡^ | 0.58 | 7.7 | 0.53 |
| 17-HDHA | 12 | 1.2 | 16 | 1.3 | 18 | 1.4 | 17 | 1.6 | 22 | 2.2 | 17 | 1.6 | 15 | 0.67 |
| 10-HDHA | 2.5 | 0.28 | 3.2 | 0.28 | 4.3 | 0.32 | 3.6 | 0.31 | 4.3 | 0.46 | 3.5 | 0.38 | 3.6 | 0.22 |
| 14-HDHA | 20 | 7.1 | 11 | 1.5 | 17 | 4.8 | 8.6 | 0.72 | 14 | 1.9 | 12 | 1.8 | 20 | 3.5 |
| 11-HDHA | 3.2 | 0.31 | 4.5 | 0.48 | 6.6^*^ | 0.38 | 5.7 | 0.69 | 7.7^†^ | 0.56 | 4.9 | 0.54 | 5.4 | 0.27 |
| 7-HDHA | 1.9 | 0.19 | 2.4 | 0.29 | 4.8^‡^ | 0.40 | 3.5 | 0.47 | 4.0^*^ | 0.40 | 2.7 | 0.28 | 2.7 | 0.10 |
| 8-HDHA | 11 | 0.71 | 13 | 1.2 | 24^‡^ | 1.0 | 20^†^ | 1.1 | 23^‡^ | 1.0 | 16 | 1.3 | 18^*^ | 0.61 |
| 4-HDHA | 14 | 1.4 | 17 | 2.6 | 21 | 1.1 | 19 | 0.94 | 28^†^ | 1.4 | 16 | 1.3 | 22 | 1.7 |
| 9,12,13-TriHOME | 44 | 11 | 50 | 12 | 24 | 2.1 | 27 | 3.5 | 24 | 1.2 | 45 | 5.5 | 69^‡^ | 27 |
| 9,10,13-TriHOME | 6.5 | 1.3 | 8.2 | 2.2 | 4.1 | 0.26 | 4.8 | 0.51 | 4.3 | 0.088 | 7.4 | 0.86 | 10 | 3.7 |
| 13-oxo-ODE | 3.7 | 0.43 | 7.4 | 1.3 | 9.2 | 0.88 | 8.5 | 1.5 | 11 | 1.7 | 6.1 | 1.2 | 8.3 | 0.24 |
| 15-oxo-ETE | 7.2 | 0.80 | 7.1 | 1.0 | 8.4 | 0.68 | 8.2 | 1.4 | 12^*^ | 1.3 | 7.5 | 1.0 | 9.3 | 0.64 |
| 9-oxo-ODE | 25 | 3.7 | 53 | 12 | 51 | 3.3 | 46 | 9.4 | 65 | 11 | 43 | 5.5 | 73 | 3.23 |
| 5-oxo-ETE | 24 | 3.4 | 27 | 2.7 | 31 | 1.4 | 28 | 3.3 | 45 | 5.3 | 17 | 3.2 | 24 | 2.9 |
| EKODE | 23 | 2.2 | 41 | 8.6 | 41 | 4.6 | 40 | 7.7 | 58 | 6.8 | 45 | 7.6 | 92^‡^ | 9.8 |
| 9(10)-EpODE | 3.5 | 0.45 | 9.1 | 1.5 | 15 | 1.9 | 13 | 2.5 | 14 | 4.5 | 8.2 | 3.0 | 9.9 | 1.1 |
| 17(18)-EpETE | 1.7 | 0.14 | 3.0 | 0.39 | 4.7 | 0.63 | 6.2^‡^ | 0.66 | 5.7^†^ | 0.56 | 2.5 | 0.66 | 2.4 | 0.25 |
| 12(13)-EpODE | 1.6 | 0.19 | 4.2 | 0.72 | 6.4 | 0.80 | 6.0 | 1.2 | 8.1^*^ | 0.84 | 3.4 | 1.1 | 4.3 | 0.37 |
| 14(15)-EpETE | 1.1 | 0.11 | 2.3 | 0.38 | 4.2^*^ | 0.56 | 5.4^‡^ | 0.54 | 4.7^†^ | 0.44 | 1.7 | 0.55 | 1.7 | 0.20 |
| 11(12)-EpETE | 1.1^*^ | 0.10 | 2.5 | 0.45 | 3.5^*^ | 0.46 | 4.4 | 0.51 | 4.0 | 0.46 | 1.5 | 0.41 | 1.4 | 0.10 |
| 8(9)-EpETE | < 1.0 | - | 1.5 | 0.14 | 2.4^*^ | 0.30 | 2.9^†^ | 0.18 | 2.8^†^ | 0.37 | < 1.0 | - | 1.2 | 0.079 |
| 19(20)-EpDPE | 31 | 2.5 | 51 | 6.5 | 78^*^ | 7.7 | 92^‡^ | 4.1 | 88^†^ | 6.2 | 43 | 7.8 | 36 | 2.3 |
| 12(13)-EpOME | 30 | 3.5 | 66 | 11 | 120^†^ | 12 | 110 | 15 | 120^*^ | 11 | 59 | 19 | 80 | 8.0 |
| 14(15)-EpETrE | 28 | 2.7 | 31 | 4.0 | 57^†^ | 5.8 | 60^†^ | 4.3 | 57^*^ | 5.9 | 28 | 8.4 | 29 | 3.6 |
| 9(10)-EpOME | 37 | 4.0 | 91 | 15 | 160^*^ | 17 | 150 | 23 | 170^*^ | 12 | 81 | 26 | 110 | 11 |
| 16(17)-EpDPE | 16 | 1.8 | 27 | 3.7 | 52^‡^ | 5.9 | 61^‡^ | 3.2 | 52^†^ | 3.6 | 23 | 6.0 | 21 | 1.8 |
| 13(14)-EpDPE | 16 | 1.8 | 28 | 3.9 | 55^†^ | 6.5 | 65^†^ | 3.4 | 56^†^ | 4.0 | 24 | 6.4 | 22 | 1.8 |
| 10(11)-EpDPE | 20 | 2.2 | 36 | 4.9 | 70^‡^ | 7.9 | 82^‡^ | 3.9 | 71^†^ | 4.5 | 30 | 8.1 | 27 | 1.9 |
| 11(12)-EpETrE | 40 | 3.8 | 41 | 4.8 | 77^†^ | 7.9 | 80^†^ | 5.5 | 82^†^ | 7.1 | 35 | 9.4 | 35 | 2.2 |
| 8(9)-EpETrE | 14 | 1.4 | 17 | 2.1 | 30^†^ | 2.7 | 32^†^ | 2.4 | 34^‡^ | 3.1 | 15 | 4.0 | 15 | 0.82 |
| 5(6)-EpETrE | 60 | 5.6 | 78 | 11 | 130^†^ | 12 | 130^*^ | 8.4 | 140^†^ | 12 | 66 | 17 | 70 | 5.6 |
| 15(16)-EpODE | 16 | 1.8 | 28 | 3.9 | 55 | 6.5 | 65 | 3.4 | 56^†^ | 4.0 | 24 | 6.4 | 22 | 1.8 |
| 9(10)-Epoxystearic acid | 29 | 2.6 | 46 | 6.1 | 81^†^ | 7.1 | 77^*^ | 6.5 | 75^*^ | 5.3 | 40 | 10 | 64 | 8.4 |
| 15,16-DiHODE | 29 | 3.2 | 26 | 2.4 | 21 | 2.1 | 13 | 1.8 | 26 | 5.3 | 20 | 1.9 | 22 | 2.3 |
| 9,10-DiHODE | 1.7 | 0.25 | 1.7 | 0.14 | 1.3 | 0.13 | 1.1 | 0.18 | 1.6 | 0.35 | 1.3 | 0.20 | 2.0 | 0.15 |
| 12,13-DiHODE | 2.6 | 0.25 | < 2.0 | - | < 2.0 | - | < 2.0 | - | < 2.0 | - | < 2.0 | - | < 2.0 | - |
| 17,18-DiHETE | 2.7^*^ | 0.074 | 3.7 | 0.20 | 2.1^‡^ | 0.049 | 2.7 | 0.28 | 4.4 | 0.63 | 2.6^*^ | 0.47 | 1.8^‡^ | 0.15 |
| 14,15-DiHETE | 0.73 | 0.027 | 1.0 | 0.050 | 0.34^‡^ | 0.026 | 0.7 | 0.12 | 1.1 | 0.20 | 0.62 | 0.13 | 0.38^‡^ | 0.030 |
| 12,13-DiHOME | 54 | 6.2 | 77 | 8.2 | 28^†^ | 2.2 | 32^*^ | 3.0 | 78 | 19 | 48 | 12 | 32^†^ | 0.62 |
| 9,10-DiHOME | 18^*^ | 2.5 | 30 | 2.9 | 17^*^ | 1.7 | 15^†^ | 1.4 | 31 | 6.2 | 22 | 3.1 | 27 | 1.5 |
| 19,20-DiHDPE | 55^*^ | 2.3 | 95 | 6.5 | 29^‡^ | 0.77 | 59 | 15 | 100 | 21 | 68 | 16 | 31^‡^ | 1.7 |
| 14,15-DiHETrE | 5.3^†^ | 0.37 | 8.0 | 0.60 | 3.3^‡^ | 0.16 | 5.9 | 0.37 | 8.5 | 0.90 | 7.1 | 1.0 | 3.4^‡^ | 0.11 |
| 16,17-DiHDPE | 9.9 | 0.55 | 14 | 1.0 | 2.8^‡^ | 0.16 | 8.2 | 2.6 | 15 | 3.6 | 8.5 | 2.4 | 2.7^‡^ | 0.18 |
| 11,12-DiHETrE | 1.7^*^ | 0.061 | 2.7 | 0.32 | 1.7^*^ | 0.085 | 2.1 | 0.12 | 2.8 | 0.31 | 2.6 | 0.36 | 1.8^‡^ | 0.07 |
| 13,14-DiHDPE | 2.6^†^ | 0.081 | 4.9 | 0.43 | 1.5^‡^ | 0.10 | 3.0 | 0.67 | 5.0 | 1.0 | 3.2 | 0.76 | 1.4 | 0.042 |
| 10,11-DiHDPE | 1.2^‡^ | 0.059 | 2.1 | 0.20 | 2.0 | 0.072 | 1.9 | 0.19 | 2.4 | 0.25 | 2.3 | 0.22 | 2.3 | 0.10 |
| 8,9-DiHETrE | 1.2 | 0.061 | 1.6 | 0.15 | 1.7 | 0.12 | 1.6 | 0.08 | 1.9 | 0.19 | 1.6 | 0.20 | 2.2^*^ | 0.14 |
| 5,6-DiHETE | < 0.25 | - | < 0.25 | - | 1.0 | 0.15 | < 0.25 | - | < 0.25 | - | < 0.25 | - | < 0.25 | - |
| 7,8-DiHDPE | 1.1^*^ | 0.038 | 1.6 | 0.11 | 1.9 | 0.10 | 1.6 | 0.10 | 2.0^*^ | 0.21 | 1.7 | 0.13 | 1.9 | 0.099 |
| 5,6-DiHETrE | 1.0 | 0.049 | 1.2 | 0.077 | 2.1^‡^ | 0.10 | 1.6^*^ | 0.12 | 1.8^†^ | 0.078 | 1.5 | 0.058 | 2.2^‡^ | 0.14 |
| 4,5-DiHDPE | 6.1^†^ | 0.66 | 13 | 0.86 | 22^‡^ | 1.2 | 15 | 1.4 | 15 | 1.2 | 15 | 0.75 | 26^‡^ | 2.4 |
| 9,10-Dihydroxystearic acid | 59 | 4.8 | 81 | 16 | 58 | 2.9 | 57 | 2.4 | 50 | 1.8 | 60 | 4.8 | 87 | 17 |

If analyte concentration is below the limit of quantification (LOQ), it is indicated as <. The given value represents the LOQ.

^*^ p <0.05 Dunnetts test vs. LPS

^†^ p <0.01 Dunnetts test vs. LPS

^‡^ p <0.001 Dunnetts test vs. LPS

**Table S4:** Oxylipin concentrations in liver tissue 24 h after LPS treatment.

| **COXi** | **-** | | **-** | | **Celecoxib** | | **Apigenin** | | **Genistein** | | **Resveratrol** | | **ε-viniferin** | |
| --- | --- | --- | --- | --- | --- | --- | --- | --- | --- | --- | --- | --- | --- | --- |
| **LPS** | **-** | | **+** | | **+** | | **+** | | **+** | | **+** | | **+** | |
| **Analyte (nM)** | **Mean** | **SE** | **Mean** | **SE** | **Mean** | **SE** | **Mean** | **SE** | **Mean** | **SE** | **Mean** | **SE** | **Mean** | **SE** |
| TXB_2_ | 25 | 7.4 | 13 | 3.1 | 12 | 4.1 | 17 | 3.1 | 31 | 5.7 | 10 | 2.0 | 8.4 | 1.6 |
| PGF_2α_ | 65 | 21 | 40 | 9.8 | 17 | 4.9 | 26 | 3.8 | 57 | 7.4 | 37 | 7.7 | 12 | 2.0 |
| PGE_2_ | 14 | 5.0 | 15 | 3.1 | 11 | 3.6 | 15 | 2.5 | 21 | 3.4 | 24 | 6.7 | 4.2 | 0.89 |
| 6-keto-PGF_1α_ | 56 | 11 | 45 | 7.1 | 32 | 11 | 32 | 7.1 | 74 | 14 | 49 | 9.5 | 29 | 5.1 |
| PGF_2α_ | 65 | 21 | 40 | 9.8 | 17 | 4.9 | 26 | 3.8 | 57 | 7.4 | 37 | 7.7 | 12 | 2.0 |
| PGE_1_ | 2.6 | 0.87 | 2.7 | 0.57 | 1.9 | 0.63 | 2.3 | 0.50 | 3.7 | 0.59 | 3.0 | 0.68 | 0.88 | 0.19 |
| PGD_1_ | 1.7 | 0.38 | 1.3 | 0.21 | 0.87 | 0.16 | 1.2 | 0.23 | 2.3 | 0.37 | 1.3 | 0.21 | 0.91 | 0.077 |
| PGD_2_ | 31 | 8.8 | 25 | 5.0 | 14 | 4.4 | 34 | 8.1 | 42 | 7.5 | 35 | 5.5 | 13 | 1.7 |
| 5-iPF_2α_ | 1.2 | 0.086 | 1.3 | 0.13 | 1.5 | 0.13 | 1.1 | 0.067 | < 1.0 | - | < 1.0 | - | 2.3^‡^ | 0.28 |
| dihomo-PGF_2α_ | 3.7 | 1.3 | 3.6 | 0.86 | 1.8 | 0.68 | 3.6 | 0.73 | 5.3 | 1.0 | 4.1 | 0.97 | 1.0 | 0.27 |
| 13,14-dihydro-15-keto-PGF_2α_ | 7.1 | 0.62 | 5.2 | 0.35 | 2.6^*^ | 0.22 | 7.0 | 1.3 | 8.2^*^ | 1.0 | 6.2 | 0.79 | 7.0 | 0.36 |
| TxB_3_ | 1.6 | 0.45 | 2.3 | 0.49 | 0.96 | 0.24 | 0.89 | 0.12 | 1.2 | 0.14 | 0.80 | 0.11 | < 0.05 | - |
| PGF_1α_ | 8.8 | 2.5 | 5.8 | 1.1 | 2.4 | 0.67 | 2.8 | 0.30 | 6.9 | 0.71 | 5.5 | 0.87 | 2.5 | 0.35 |
| 13,14-dihydro-15-keto-PGE_1_ | 0.63 | 0.050 | 0.38 | 0.042 | 0.31 | 0.070 | 0.57 | 0.23 | 0.61 | 0.078 | 0.53 | 0.050 | 0.62 | 0.13 |
| 8-iPF_2α_ | 1.6 | 0.65 | 0.62 | 0.22 | 1.4 | 0.41 | 2.1 | 0.11 | 3.0^*^ | 0.52 | 2.2 | 0.71 | < 0.10 | - |
| 9-HOTrE | 20^†^ | 2.5 | 11 | 1.3 | 9.0 | 0.93 | 8.1 | 1.1 | 9.9 | 1.3 | 9.3 | 1.0 | 5.0 | 0.58 |
| 13-HOTrE | 110 | 50 | 21 | 4.9 | 11 | 1.8 | 18 | 4.5 | 18 | 5.9 | 19 | 5.0 | 8.5 | 1.5 |
| 20-HETE | < 2.6 | - | 5.3 | 0.67 | 4.5 | 0.67 | 7.2 | 2.2 | 6.3 | 1.7 | 4.1 | 0.18 | < 2.6 | - |
| 15-HEPE | 11 | 3.3 | 4.2 | 0.82 | 2.2 | 0.25 | 3.4 | 0.86 | 4.0 | 1.0 | 3.2 | 0.52 | 1.7 | 0.27 |
| 8-HEPE | 1.2 | 0.17 | 0.99 | 0.048 | 0.84 | 0.094 | 0.98 | 0.23 | 1.2 | 0.089 | 1.0 | 0.16 | < 0.60 | - |
| 12-HEPE | 85 | 28 | 40 | 9.2 | 19 | 2.7 | 27 | 11 | 39 | 6.1 | 19 | 4.9 | 6.8 | 1.1 |
| 5-HEPE | 3.0 | 0.28 | 3.0 | 0.42 | 4.6 | 0.72 | 2.8 | 0.80 | 2.7 | 0.37 | 2.3 | 0.18 | 1.2 | 0.087 |
| 13-HODE | 1400 | 550 | 500 | 72 | 300 | 29 | 370 | 79 | 530 | 98 | 520 | 48 | 230 | 15 |
| 9-HODE | 600 | 79 | 480 | 58 | 300 | 36 | 370 | 64 | 550 | 85 | 540 | 38 | 250 | 16 |
| 15-HETE | 42 | 9.0 | 32 | 3.5 | 26 | 2.1 | 31 | 6.0 | 40 | 5.4 | 34 | 4.5 | 14 | 0.86 |
| 11-HETE | 24 | 5.8 | 21 | 3.0 | 13 | 2.2 | 21 | 4.0 | 35 | 5.1 | 23 | 3.3 | 7.0 | 0.93 |
| 8-HETE | 13 | 2.7 | 8.2 | 0.79 | 7.4 | 0.86 | 8.5 | 1.9 | 10 | 1.4 | 8.2 | 1.6 | 4.2 | 0.33 |
| 12-HETE | 430^*^ | 122 | 140 | 29 | 98 | 15 | 120 | 50 | 150 | 21 | 69 | 17 | 32 | 6.0 |
| 9-HETE | 4.3 | 0.54 | 4.5 | 0.41 | 6.2 | 0.71 | 5.2 | 1.1 | 6.2 | 0.84 | 6.3 | 0.91 | 2.9 | 0.12 |
| 15(S)-HETrE | 18 | 4.1 | 11 | 1.1 | 8.9 | 0.98 | 9.0 | 1.4 | 14 | 1.4 | 11 | 1.2 | 5.2 | 0.37 |
| 5-HETE | 3.0 | 0.28 | 3.0 | 0.42 | 4.6^†^ | 0.72 | 2.8 | 0.80 | 2.7 | 0.37 | 2.3 | 0.18 | 1.2 | 0.087 |
| 20-HEPE | 18 | 2.1 | 25 | 1.8 | 5.9^‡^ | 0.72 | 15 | 2.4 | 22 | 5.8 | 16 | 3.2 | 4.1^‡^ | 0.51 |
| 18-HEPE | 3.4 | 0.50 | 3.3 | 0.20 | 3.1 | 0.25 | 3.3 | 0.73 | 4.6 | 0.53 | 4.1 | 0.26 | 1.6 | 0.17 |
| 20-HDHA | 13 | 1.5 | 18 | 1.2 | 20 | 1.4 | 22 | 3.5 | 27^*^ | 2.7 | 22 | 1.7 | 9.1^*^ | 0.83 |
| 16-HDHA | 4.2 | 0.50 | 5.0 | 0.35 | 5.2 | 0.70 | 6.0 | 1.1 | 8.1^*^ | 0.80 | 6.3 | 0.74 | 2.4 | 0.25 |
| 13-HDHA | 7.8 | 1.2 | 8.5 | 0.82 | 8.3 | 0.84 | 10 | 2.1 | 16^†^ | 2.0 | 10 | 1.2 | 3.8 | 0.48 |
| 17-HDHA | 49 | 14 | 22 | 3.4 | 18 | 2.4 | 21 | 4.9 | 25 | 4.4 | 19 | 2.7 | 9.0 | 0.90 |
| 10-HDHA | 6.3 | 1.0 | 5.1 | 0.48 | 5.5 | 0.59 | 5.7 | 1.3 | 7.6 | 0.56 | 4.9 | 0.58 | 2.2 | 0.15 |
| 14-HDHA | 240^*^ | 73 | 70 | 14 | 60 | 8.0 | 68 | 21 | 97 | 13 | 48 | 9.4 | 18 | 2.9 |
| 11-HDHA | 4.0 | 0.47 | 4.4 | 0.39 | 7.0 | 1.1 | 5.5 | 0.94 | 7.0 | 0.82 | 5.0 | 0.46 | 2.8 | 0.31 |
| 7-HDHA | 2.4 | 0.28 | 2.8 | 0.26 | 5.6^‡^ | 0.50 | 3.2 | 0.64 | 4.4 | 0.64 | 2.7 | 0.37 | 1.7 | 0.15 |
| 8-HDHA | 12 | 1.2 | 15 | 1.1 | 24^*^ | 3.3 | 20 | 3.6 | 25^*^ | 2.9 | 18 | 1.2 | 9.0 | 0.93 |
| 4-HDHA | 7.6 | 0.92 | 10 | 0.83 | 18^†^ | 1.7 | 12 | 2.7 | 14 | 1.4 | 13 | 1.4 | 6.8 | 0.65 |
| 9,12,13-TriHOME | 230 | 48 | 190 | 39 | 77 | 16 | 41 | 3.0 | 64 | 11 | 300 | 63 | 110 | 26 |
| 9,10,13-TriHOME | 26 | 3.3 | 27 | 5.5 | 9.2^*^ | 1.1 | 6.4^*^ | 0.76 | 8.6^*^ | 1.1 | 46 | 8.0 | 16 | 3.5 |
| 13-oxo-ODE | 13 | 2.7 | 17 | 2.2 | 12 | 1.7 | 16 | 2.2 | 20 | 3.2 | 23 | 1.5 | 9.4 | 0.86 |
| 15-oxo-ETE | 3.3 | 0.65 | 4.2 | 0.18 | 6.8 | 0.89 | 4.0 | 1.4 | 5.8 | 0.96 | 5.5 | 1.1 | 2.8 | 0.23 |
| 9-oxo-ODE | 82 | 18 | 120 | 15 | 66 | 12 | 120 | 14 | 160 | 26 | 150 | 8.5 | 53 | 3.9 |
| 5-oxo-ETE | 13 | 2.2 | 11 | 0.53 | 35 | 2.8 | 15 | 2.6 | 19 | 3.46 | 17 | 2.8 | 14 | 2.4 |
| EKODE | 40 | 11 | 45 | 3.7 | 70 | 16 | 28 | 9.5 | 40 | 6.78 | 53 | 6.0 | 34 | 4.9 |
| 9(10)-EpODE | 8.2 | 1.5 | 10 | 1.0 | 15 | 1.7 | 11 | 2.7 | 12 | 1.81 | 12 | 1.1 | 9.2 | 0.70 |
| 17(18)-EpETE | 3.6 | 0.43 | 3.8 | 0.37 | 5.0 | 0.37 | 4.2 | 0.77 | 4.6 | 0.44 | 4.8 | 0.45 | 3.2 | 0.30 |
| 12(13)-EpODE | 3.9 | 0.69 | 4.9 | 0.46 | 6.2 | 0.73 | 5.0 | 1.1 | 5.7 | 1.01 | 5.8 | 0.48 | 3.9 | 0.49 |
| 14(15)-EpETE | 2.2 | 0.32 | 2.5 | 0.18 | 3.5 | 0.30 | 3.1 | 0.56 | 3.3 | 0.3 | 3.2 | 0.32 | 1.8 | 0.25 |
| 11(12)-EpETE | 2.3 | 0.33 | 2.8 | 0.23 | 3.3 | 0.35 | 4.4 | 0.55 | 5.1^†^ | 0.47 | 4.6^*^ | 0.46 | 2.0 | 0.20 |
| 8(9)-EpETE | 1.5 | 0.23 | 1.5 | 0.17 | 2.5^*^ | 0.26 | 2.1 | 0.34 | 2.5 | 0.21 | 2.3 | 0.32 | 1.5 | 0.12 |
| 19(20)-EpDPE | 22 | 2.4 | 25 | 1.8 | 47^‡^ | 5.3 | 33 | 3.9 | 40^*^ | 1.9 | 33 | 3.8 | 17 | 1.8 |
| 12(13)-EpOME | 68 | 9.7 | 82 | 8.3 | 120 | 14 | 99 | 12 | 110 | 12 | 110 | 16 | 72 | 3.3 |
| 14(15)-EpETrE | 26 | 3.8 | 26 | 2.1 | 51^†^ | 6.5 | 38 | 4.3 | 44^*^ | 4.4 | 33 | 5.3 | 21 | 2.0 |
| 9(10)-EpOME | 82 | 12 | 97 | 9.3 | 140 | 16 | 120 | 17 | 130 | 15 | 120 | 12 | 84 | 5.7 |
| 16(17)-EpDPE | 14 | 1.8 | 16 | 1.3 | 31^‡^ | 4.2 | 23 | 3.0 | 27^*^ | 1.3 | 23 | 2.8 | 11 | 1.4 |
| 13(14)-EpDPE | 14 | 1.9 | 16 | 1.3 | 32^‡^ | 4.0 | 24 | 2.9 | 27^*^ | 1.4 | 23 | 3.0 | 12 | 1.6 |
| 10(11)-EpDPE | 17 | 2.3 | 20 | 1.7 | 40^‡^ | 5.1 | 30 | 3.8 | 33^*^ | 1.8 | 28 | 3.4 | 15 | 1.9 |
| 11(12)-EpETrE | 34 | 4.6 | 32 | 2.4 | 58^†^ | 6.3 | 47 | 7.0 | 51 | 5.3 | 39 | 6.3 | 27 | 2.9 |
| 8(9)-EpETrE | 13 | 2.0 | 13 | 1.0 | 25^†^ | 2.8 | 20 | 2.8 | 20 | 2.3 | 14 | 1.8 | 9.8 | 0.91 |
| 5(6)-EpETrE | 59 | 9.0 | 59 | 3.2 | 120^†^ | 13 | 97 | 16 | 90 | 12 | 64 | 7.8 | 45 | 4.3 |
| 15(16)-EpODE | 14 | 1.9 | 16 | 1.3 | 32 | 4.0 | 24 | 2.9 | 27 | 1.4 | 23 | 3.0 | 12 | 1.6 |
| 9(10)-Epoxystearic acid | 61 | 4.6 | 61 | 3.4 | 75 | 10 | 86 | 9.4 | 82 | 6.1 | 84 | 11 | 66 | 8.6 |
| 15,16-DiHODE | 150 | 18 | 120 | 23 | 47^*^ | 7.1 | 49 | 7.6 | 63 | 13 | 49 | 6.9 | 17^†^ | 1.7 |
| 8,15-DiHETE | 3.8 | 1.9 | < 0.80 | - | < 0.80 | - | < 0.80 | - | 0.88 | 0.067 | < 0.80 | - | < 0.80 | - |
| 9,10-DiHODE | 13 | 1.6 | 10 | 1.8 | 4.4 | 0.58 | 8.6 | 2.3 | 7.6 | 1.3 | 6.1 | 1.4 | 3.9 | 0.55 |
| 12,13-DiHODE | 20 | 3.2 | 13 | 3.5 | 3.2 | 0.56 | 5.7 | 1.4 | 5.7 | 1.3 | 6.1 | 1.5 | < 2.0 | - |
| 17,18-DiHETE | 22 | 2.2 | 23 | 1.5 | 9.4^†^ | 0.81 | 15 | 2.3 | 19 | 4.0 | 18 | 3.3 | 5.6^‡^ | 0.54 |
| 14,15-DiHETE | 12 | 1.1 | 10 | 0.51 | 2.6^‡^ | 0.18 | 7.4 | 1.2 | 8.6 | 2.1 | 8.1 | 2.0 | 2.1^‡^ | 0.21 |
| 11,12-DiHETE | 3.6 | 0.34 | 3.0 | 0.19 | 1.2^*^ | 0.078 | 2.9 | 0.67 | 2.9 | 0.62 | 2.9 | 0.75 | 1.0^*^ | 0.11 |
| 12,13-DiHOME | 370 | 32 | 340 | 31 | 70^‡^ | 8.3 | 140^†^ | 17 | 210 | 60 | 200 | 53 | 45^‡^ | 2.6 |
| 8,9-DiHETE | 2.8 | 0.33 | 2.4 | 0.15 | 1.4 | 0.13 | 2.3 | 0.70 | 2.6 | 0.52 | 2.4 | 0.48 | 1.2 | 0.13 |
| 9,10-DiHOME | 93 | 6.9 | 95 | 7.6 | 40^†^ | 2.8 | 69 | 16 | 82 | 16 | 79 | 17 | 43^*^ | 4.6 |
| 19,20-DiHDPE | 150 | 22 | 200 | 15 | 55^†^ | 5.9 | 110 | 18 | 170 | 49 | 140 | 35 | 17^‡^ | 3.6 |
| 14,15-DiHETrE | 71 | 9.9 | 67 | 4.5 | 23^†^ | 1.4 | 43 | 6.9 | 62 | 14 | 47 | 11 | 12^‡^ | 1.1 |
| 16,17-DiHDPE | 32 | 3.5 | 38 | 2.4 | 8.0^‡^ | 0.55 | 22 | 4.1 | 35 | 9.9 | 27 | 6.4 | 3.0^‡^ | 0.36 |
| 11,12-DiHETrE | 26 | 3.8 | 24 | 1.8 | 11^*^ | 0.53 | 18 | 3.5 | 22 | 4.3 | 17 | 4.0 | 5.5^†^ | 0.68 |
| 13,14-DiHDPE | 11 | 1.4 | 15 | 1.1 | 4.4^†^ | 0.28 | 11 | 2.0 | 15 | 3.8 | 11 | 2.2 | 1.6^‡^ | 0.20 |
| 10,11-DiHDPE | 8.2 | 1.2 | 10 | 0.87 | 4.8^*^ | 0.37 | 9.8 | 2.1 | 11 | 2.3 | 7.8 | 1.4 | 2.2^†^ | 0.27 |
| 8,9-DiHETrE | 12 | 1.8 | 11 | 0.96 | 7.9 | 0.51 | 10 | 2.8 | 12 | 2.3 | 8.7 | 1.8 | 4.4 | 0.56 |
| 5,6-DiHETE | < 0.30 | - | < 0.30 | - | 0.99 | 0.21 | < 0.30 | - | < 0.30 | - | < 0.30 | - | < 0.30 | - |
| 7,8-DiHDPE | 4.8 | 0.62 | 5.2 | 0.47 | 3.8 | 0.33 | 5.2 | 1.2 | 6.3 | 1.0 | 4.5 | 0.73 | 1.9 | 0.23 |
| 5,6-DiHETrE | 4.2 | 0.72 | 3.9 | 0.32 | 4.9 | 0.42 | 5.1 | 1.5 | 6.3 | 1.1 | 4.0 | 0.73 | 1.9 | 0.16 |
| 4,5-DiHDPE | 44 | 12 | 57 | 6.8 | 76 | 7.9 | 74 | 18 | 64 | 11 | 70 | 5.8 | 20 | 4.6 |
| 9,10-Dihydroxystearic acid | 140 | 19 | 160 | 36 | 65 | 2.8 | 53 | 2.2 | 64 | 3.7 | 260 | 28 | 170 | 21 |

If analyte concentration is below the limit of quantification (LOQ), it is indicated as <. The given value represents the LOQ.

^*^ p <0.05 Dunnetts test vs. LPS

^†^ p <0.01 Dunnetts test vs. LPS

^‡^ p <0.001 Dunnetts test vs. LPS

**Table S5:** Effect of commonly used anti-inflammatory drugs on COX activity under the same assay conditions [18].

|  | cell-free – COX-1 | cell-free – COX-2 | HCA-7 | | primary monocytes | |
| --- | --- | --- | --- | --- | --- | --- |
|  | **IC_50_ (nM) ^*^**  (95% CI) | **IC_50_ (nM) ^*^**  (95% CI) | **IC_50_ (nM) ^*^**  (95% CI) | **COX-2**  **Expression ^†^** | **IC_50_ (nM) ^*^**  (95% CI) | **COX-2**  **Expression ^†^** |
| **celecoxib** | **21500**  (17400-26700) | **242**  (102-578) | **292**  (179-477) | no effect  (up to 10 µM) | **14**  (8.0-24) | no effect  (up to 1 µM) |
| **indomethacin** | 17  (11-27) | **362**  (195-671) | **583**  (254-1360) | no effect  (up to 25 µM) | **10**  (6.7-16) | no effect  (up to 100 µM) |
| **dexamethasone** | no effect  (up to 100 µM) | no effect  (up to 100 µM) | no effect  (up to 10 µM) | no effect  (up to 100 µM) | **1.6**  (1.4-1.9) | 1 µM - 3 nM:  COX-2 ↓ |

^*^ IC_50_ values were calculated based on the PGE_2_ formation (n=3).

^†^ COX-2 protein levels were analyzed by a COX-2-specific Western Blot; a decreased COX-2 protein level in comparison to the control is indicated by ↓.

**Table S6:** Intensity of COX-2 bands shown in the western blots in figure 1. The ratio of the intensity of the COX-2 band and β-actin band are shown as % of control.

| **resveratrol** | | | | |
| --- | --- | --- | --- | --- |
| **HCA-7 cells** | |  | **monocytes** | |
| conc. (µM) | % of control |  | conc. (µM) | % of control |
| 0 (control) | 100 |  | 0 (control) | 100 |
| 0.001 | 105 |  | 0.1 | 152 |
| 0.01 | 157 |  | 1 | 142 |
| 0.1 | 160 |  | 3 | 141 |
| 10 | 139 |  | 10 | 107 |
| 50 | 101 |  | 50 | 69 |

| **apigenin** | | | | |
| --- | --- | --- | --- | --- |
| **HCA-7 cells** | |  | **monocytes** | |
| conc. (µM) | % of control |  | conc. (µM) | % of control |
| 0 (control) | 100 |  | 0 (control) | 100 |
| 0.001 | 72 |  | 0.1 | 104 |
| 0.01 | 50 |  | 1 | 22 |
| 0.1 | 70 |  | 3 | 27 |
| 1 | 255 |  | 10 | 26 |
| 10 | 100 |  | 50 | 13 |
| 50 | 89 |  |  |  |
