# Supplementary material for: Food Polyphenols Fail to Cause a Biologically Relevant Reduction of COX-2 Activity
Source: PLoS One. 2015 Oct 6;10(10):e0139147. doi: 10.1371/journal.pone.0139147 (PMC4594923; doi:10.1371/journal.pone.0139147)
Supplement: S5 Table — (DOCX) [file pone.0139147.s006.docx]

**Table S5:** Effect of commonly used anti-inflammatory drugs on COX activity under the same assay conditions [18].

|  | cell-free – COX-1 | cell-free – COX-2 | HCA-7 | | primary monocytes | |
| --- | --- | --- | --- | --- | --- | --- |
|  | **IC_50_ (nM) ^*^**  (95% CI) | **IC_50_ (nM) ^*^**  (95% CI) | **IC_50_ (nM) ^*^**  (95% CI) | **COX-2**  **Expression ^†^** | **IC_50_ (nM) ^*^**  (95% CI) | **COX-2**  **Expression ^†^** |
| **celecoxib** | **21500**  (17400-26700) | **242**  (102-578) | **292**  (179-477) | no effect  (up to 10 µM) | **14**  (8.0-24) | no effect  (up to 1 µM) |
| **indomethacin** | 17  (11-27) | **362**  (195-671) | **583**  (254-1360) | no effect  (up to 25 µM) | **10**  (6.7-16) | no effect  (up to 100 µM) |
| **dexamethasone** | no effect  (up to 100 µM) | no effect  (up to 100 µM) | no effect  (up to 10 µM) | no effect  (up to 100 µM) | **1.6**  (1.4-1.9) | 1 µM - 3 nM:  COX-2 ↓ |

^*^ IC_50_ values were calculated based on the PGE_2_ formation (n=3).

^†^ COX-2 protein levels were analyzed by a COX-2-specific Western Blot; a decreased COX-2 protein level in comparison to the control is indicated by ↓.
