# Supplementary material for: Food Polyphenols Fail to Cause a Biologically Relevant Reduction of COX-2 Activity
Source: PLoS One. 2015 Oct 6;10(10):e0139147. doi: 10.1371/journal.pone.0139147 (PMC4594923; doi:10.1371/journal.pone.0139147)
Supplement: S6 Table — The ratio of the intensity of the COX–2 band and β-actin band are shown as % of control. (DOCX) [file pone.0139147.s007.docx]

**Table S6:** Intensity of COX-2 bands shown in the western blots in figure 1. The ratio of the intensity of the COX-2 band and β-actin band are shown as % of control.

| **resveratrol** | | | | |
| --- | --- | --- | --- | --- |
| **HCA-7 cells** | |  | **monocytes** | |
| conc. (µM) | % of control |  | conc. (µM) | % of control |
| 0 (control) | 100 |  | 0 (control) | 100 |
| 0.001 | 105 |  | 0.1 | 152 |
| 0.01 | 157 |  | 1 | 142 |
| 0.1 | 160 |  | 3 | 141 |
| 10 | 139 |  | 10 | 107 |
| 50 | 101 |  | 50 | 69 |

| **apigenin** | | | | |
| --- | --- | --- | --- | --- |
| **HCA-7 cells** | |  | **monocytes** | |
| conc. (µM) | % of control |  | conc. (µM) | % of control |
| 0 (control) | 100 |  | 0 (control) | 100 |
| 0.001 | 72 |  | 0.1 | 104 |
| 0.01 | 50 |  | 1 | 22 |
| 0.1 | 70 |  | 3 | 27 |
| 1 | 255 |  | 10 | 26 |
| 10 | 100 |  | 50 | 13 |
| 50 | 89 |  |  |  |
